# Supplementary material for: Enhancing the gravity model for commuters: Time-and-spatial-structure-based improvements in Japan’s metropolitan areas
Source: PLoS One. 2025 Aug 12;20(8):e0329603. doi: 10.1371/journal.pone.0329603 (PMC12342275; doi:10.1371/journal.pone.0329603)
Supplement: S1 File — The contents are as follows: (1) Study area; (2) Commuter trajectory; (3) Commuting distance and time; (4) Deviation of the gravity model; (5) Prediction of the SSUG model; (6) Commuting propensities; (7) Regional analysis; (8) Robustness analysis; (9) Data bias; (10) CES form of the gravity model; (11) Clarification of Constraint 2 to Eq 4; (12) OD flow and commuting drivers; (13) Objective function values; (14) Clearer subplots for Fig 4. (PDF) [file pone.0329603.s001.pdf]

Enhancing the gravity model for commuters: Time-and-spatial-structure-based improvements in Japan's metropolitan areas

Supplementary Material

Yixuan Y Zheng[1], *Yohei Shida*[2,3], *Hideki Takayasu*[3], *Misako Takayasu*[1,3*]

[1] *Department of Systems and Control Engineering, School of Engineering*
Institute of Sience Tokyo, Yokohama 226-8502, Japan

[2] *Institute of Systems and Information Engineering*
University of Tsukuba, Tsukuba 305-8577, Japan
Tokyo 141-0022, Japan

[3] *Department of Computer Science, School of Computing*
Institute of Sience Tokyo, Yokohama 226-8502, Japan

Contents

1 Study area **2**

2 Commuter Trajectory **2**

3 Commuting distance and time **4**

4 Deviation of the gravity model **4**

5 Prediction of the SSUG model **5**

6 Commuting Propensities **9**

7 Region Analysis **12**

8 Robustness Analysis **15**

9 Data bias **16**

10 CES form of the gravity model **16**

11 Clarification of Constraint 2 to (Eq. 4) **17**

12 OD flow and commuting driver **18**

13 Objective Function Values **19**

14 Clearer subplots for Figure 4 **19**

1 Study area

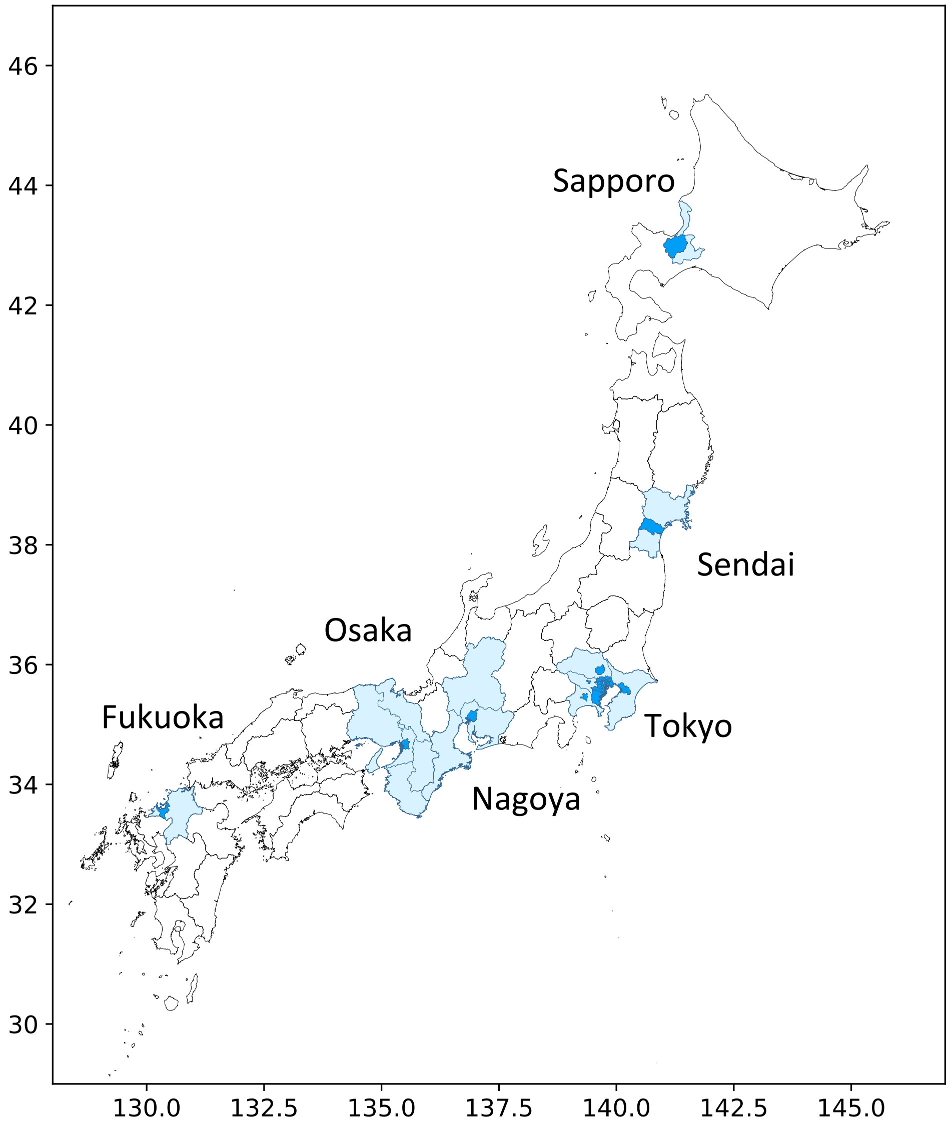

Figure S1: The map layout displays key metropolitan areas with their respective prefectures and center cities highlighted in Japan. Specifically, the selected Japanese metropolitan areas, listed as (A) Tokyo, (B) Osaka, (C) Nagoya, (D) Fukuoka, (E) Sapporo, and (F) Sendai, have been shaded in light blue. The center cities shaded in darker blue in (B) through (F) share the same names as their respective metropolitan areas. However, in the case of (A) Tokyo metropolitan area, the center city or region encompasses the main 23 districts in Tokyo. In each subplot, the x- and y-axes represent latitude and longitude, respectively. Base map reprinted from [2] under a CC BY license, with permission from Ministry of Land, Infrastructure, Transport and Tourism, original copyright 2008.

2 Commuter Trajectory

To demonstrate the reliability of ”home” and ”work” status’ identification, we present a typical commuter’s trajectory, as depicted below (Figure S2):

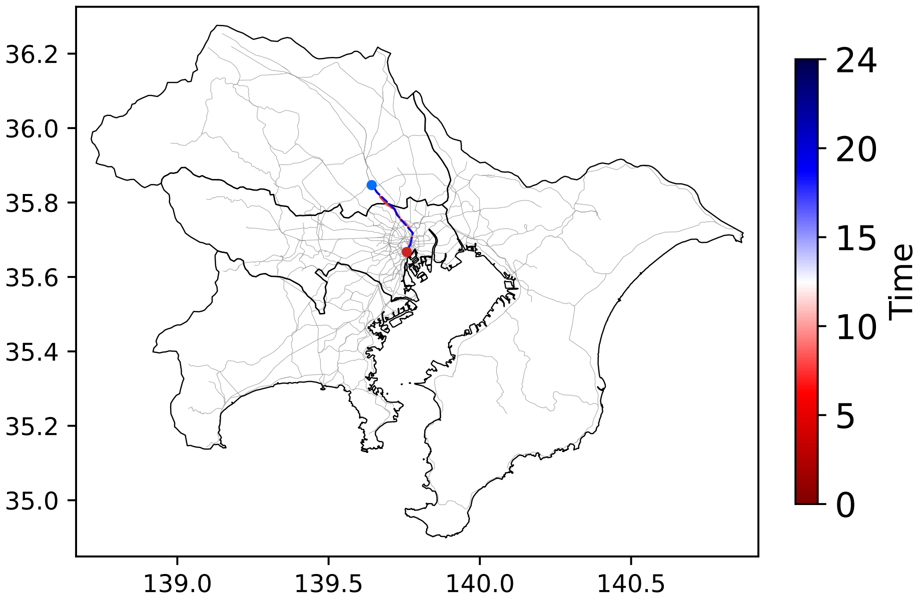

(a)

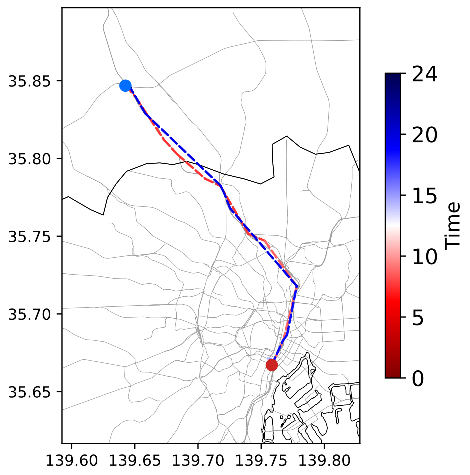

(b)

Figure S2: Map of a typical commuter's trajectory. Figures S2a and S2b illustrate a commuter's trajectory covering the entire Tokyo metropolitan area and its magnified view, respectively. Blue points on the map represent records marked as home status, whereas red points indicate works status records. The color of the trajectory lines signifies the time of day; this transition is indicated by the color bar, shifting from red (0 a.m.) to blue (23:59 p.m.). The trajectory map effectively captures the commuter's daily routine of leaving home in the morning and returning in the evening. This commuter's route overlaps with the shapes of railways, suggesting the potential use of underground transportation. The x- and y-axes correspond to latitude and longitude, respectively. Base map reprinted from [2] under a CC BY license, with permission from Ministry of Land, Infrastructure, Transport and Tourism, original copyright 2008.

3 Commuting distance and time

As described in Section 3.1.2, we defined commuting behavior as a commuter's journey from their estimated accommodation to their stable office location. This definition prioritizes commuting time over distance due to its greater sensitivity in estimation. While commuters can optimize routes, journey times remain less predictable. We note potential overestimation of commuting time for night workers due to possible lack of GPS signals during sleep hours. To maintain consistency, our analysis only included commuting behaviors lasting less than 2 hours.

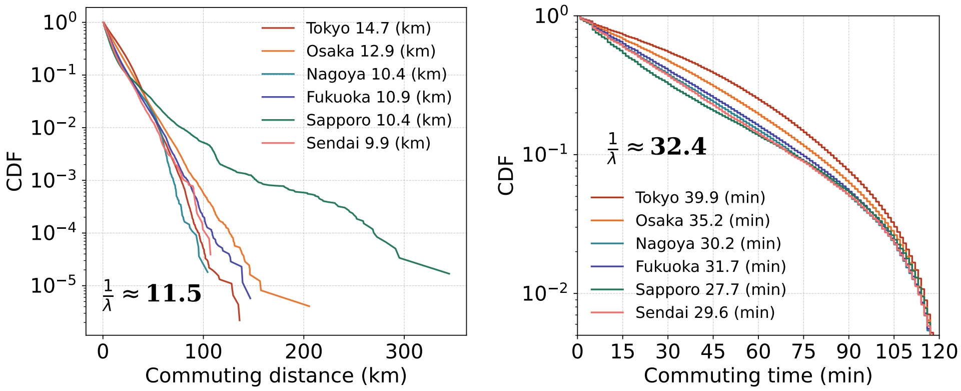

(a) (b)

Figure S3: Semi-log plots depict the cumulative distribution functions of home-based commuting distance (Fig. 3a) and time (Fig. 3b) for all the selected regions. We only considered cases with commuting times of less than 120 minutes, based on practical scenarios. The suitability of this criterion can be inferred from the original version in Supplementary Figure S3(a). The exponent for the average commuting times in the Japanese main metropolitan areas is approximately $\frac{1}{\lambda_d} \approx 11.5$ km, as shown in Fig. 3a. Similarly, for effective commuting times, it is approximately $\frac{1}{\lambda_t} \approx 32.4$ min (Fig. 3b).

4 Deviation of the gravity model

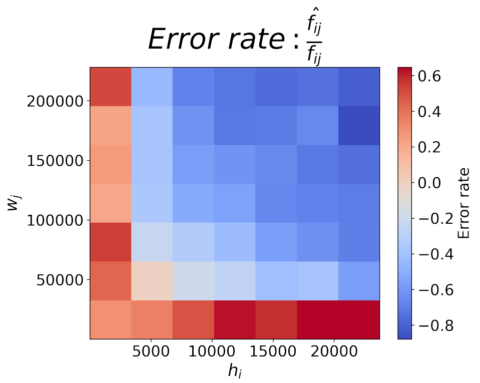

Figure S4: Deviation of the gravity model predictions compared to the actual commuting flows. The heatmap illustrates the error rate $\frac{\hat{f}_{ij}}{f_{ij}}$ as a function of the residential population (h_i) and the workplace population (w_j) scales. The blue areas indicate underestimation (error rate < 0), while the red areas signify overestimation (error rate > 0). This result suggests that the gravity model's prediction accuracy is highly dependent on the population scales of the origin and destination regions. The model tends to overestimate commuting flows for smaller population scales and underestimate them for larger population scales.

5 Prediction of the SSUG model

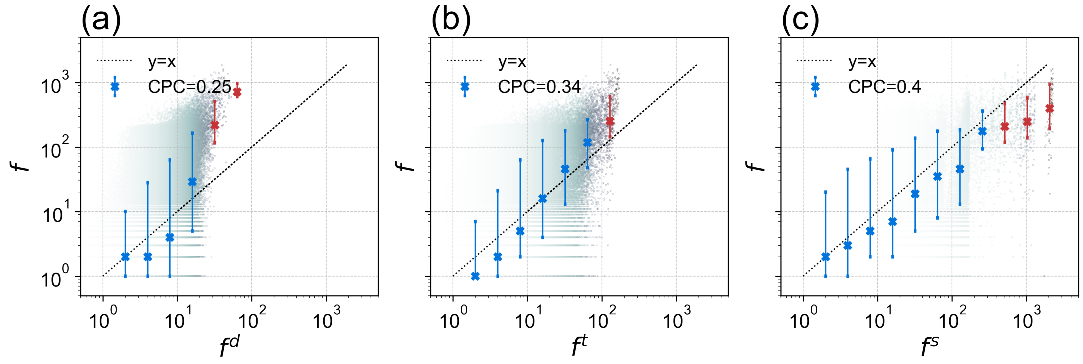

(a) Osaka

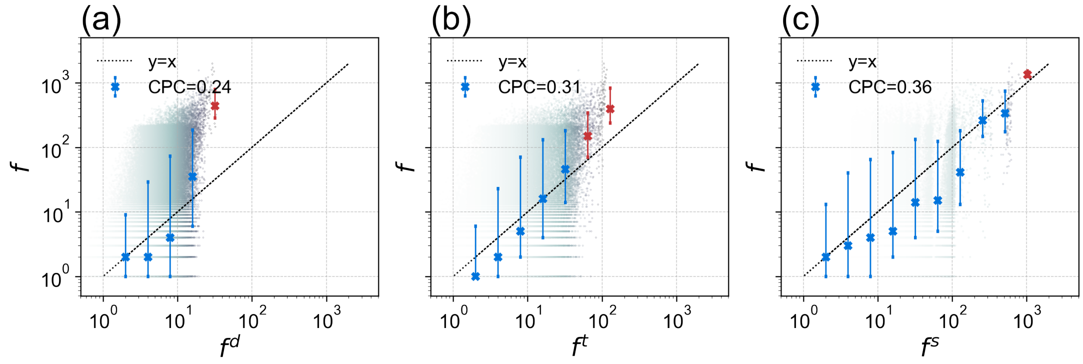

(b) Nagoya

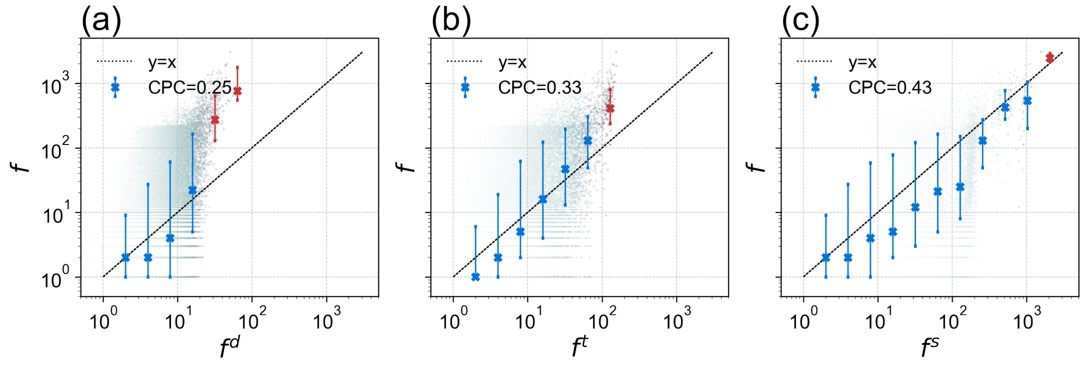

(c) Fukuoka

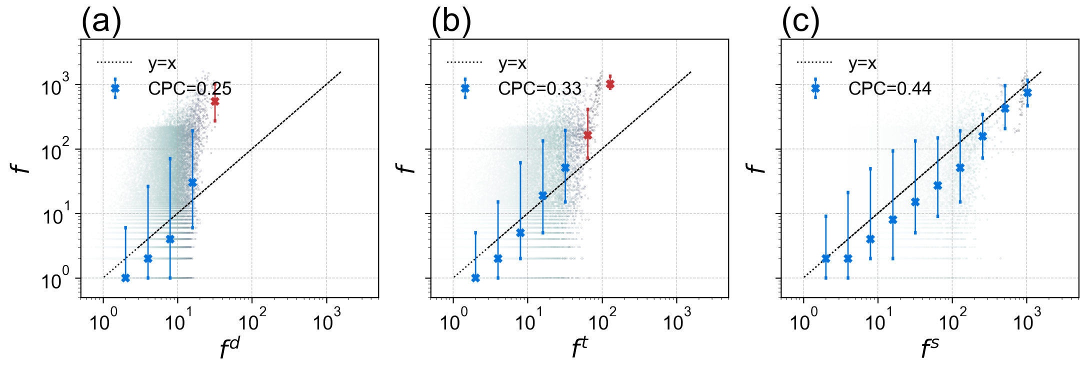

(d) Sapporo

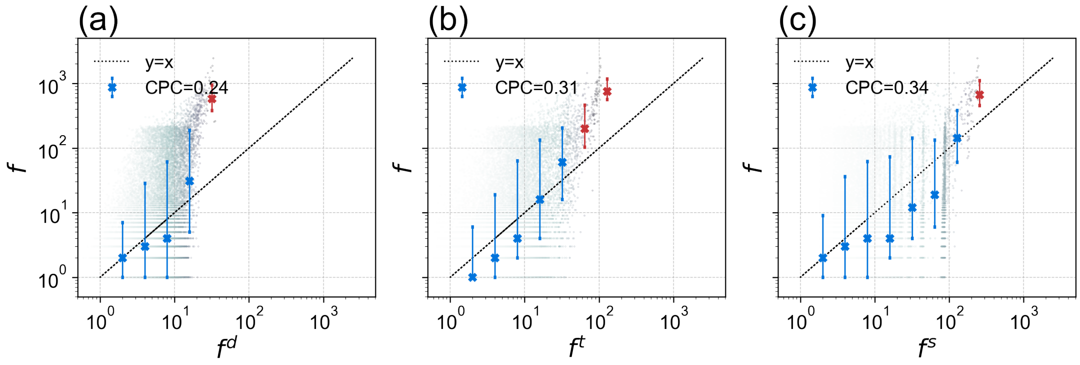

(e) Sendai

Figure S5: Overview of commuting patterns across different metropolitan areas in Japan. Each row represents a different region, displaying the performance of three models: (a) Euclidean distance-based gravity model (f^d), (b) commuting-time-based gravity model (f^t), and (c) SSUG model (f^s). The regions are presented in the following order: Osaka, Nagoya, Fukuoka, Sapporo, and Sendai. Each subplot compares the empirical commuting flows (f) observed from the data with the predicted commuting flows from the respective model. The x-axis represents the predicted flows, while the y-axis shows the actual flows. The scatter points are divided into bins based on the predicted values, with markers indicating the median real flow in each bin. Error bars show the range between the 1st and 3rd quartiles within each bin. The black dotted line ($y = x$) represents perfect prediction. Blue error bars indicate bins where the y-values fall within the 1st and 3rd quartiles of the x-values' bins, while red error bars signify significant misestimation. The CPC (Common Part of Commuters) value is provided for each model in each region, offering a quantitative measure of model performance.

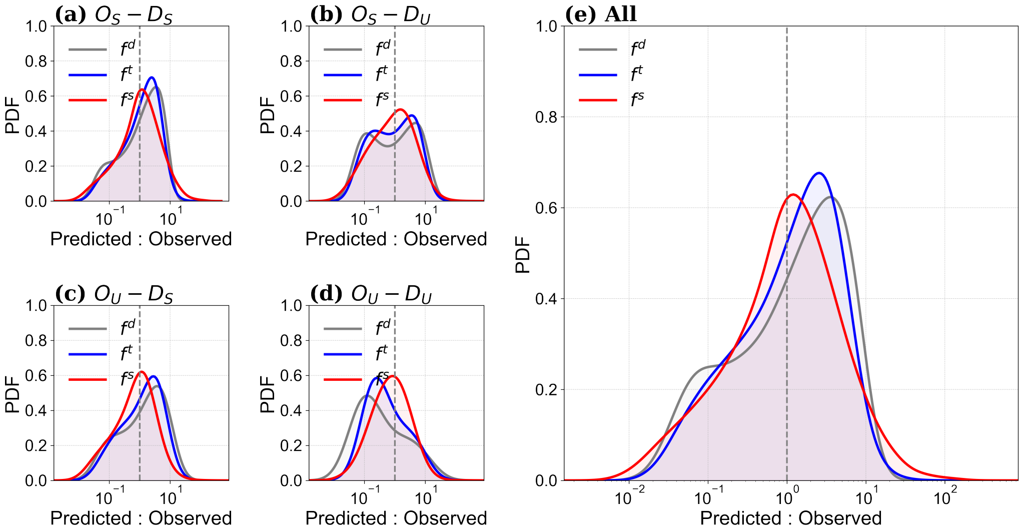

(a) Osaka

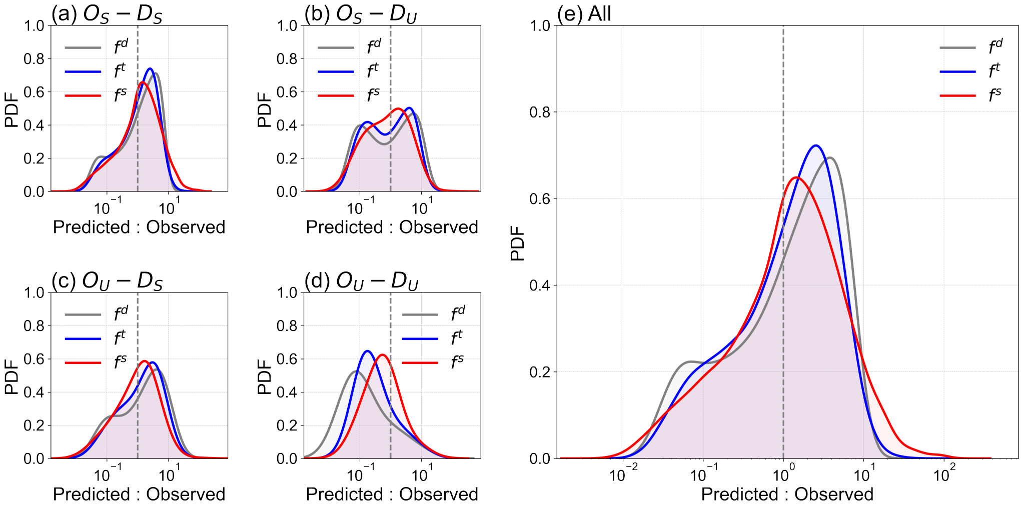

(b) Nagoya

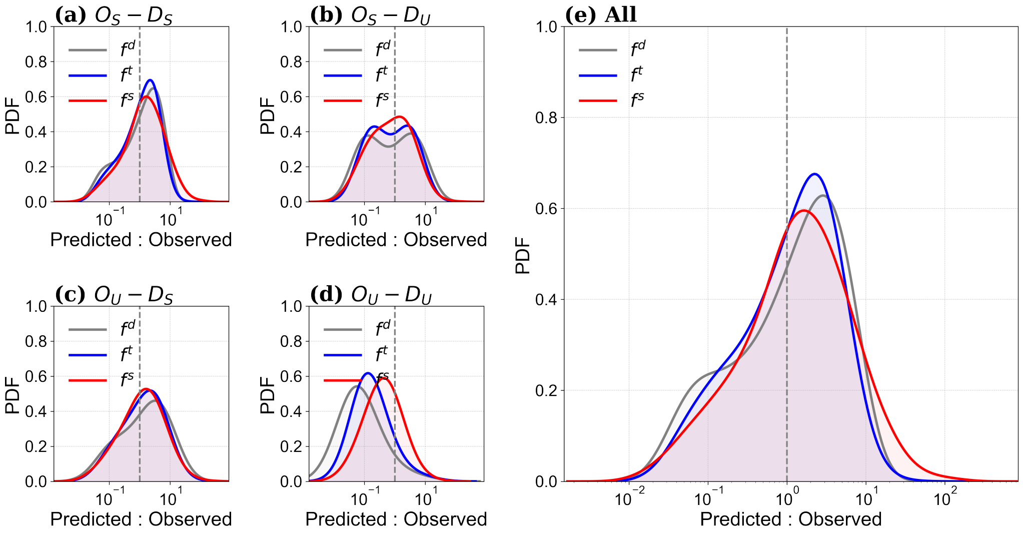

(c) Fukuoka

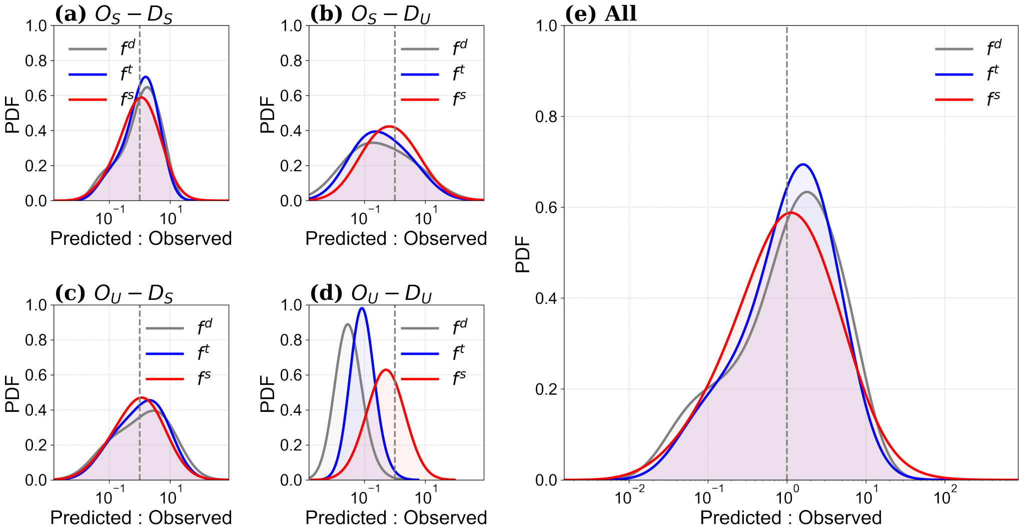

(d) Sapporo

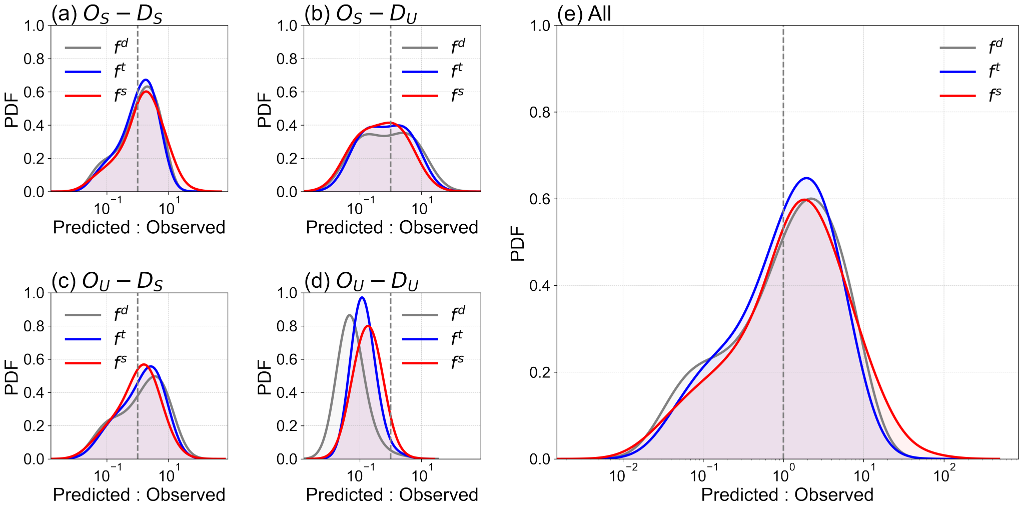

(e) Sendai

Figure S6: Overview of commuting patterns across different metropolitan areas in Japan. Each row represents a different region (Osaka, Nagoya, Fukuoka, Sapporo, and Sendai), displaying the probability density function (PDF) of the ratio between predicted and observed trip counts for different commuting trip types. The comparison is made between three models: the distance-based gravity model (f^d, grey), the commuting time-based gravity model (f^t, blue), and the SSUG model (f^s, red). Subplots (a)–(d) in each row depict the PDF for specific trip types: (a) O_s–D_s, (b) O_s–D_u, (c) O_u–D_s, and (d) O_u–D_u, where O and D represent origin and destination, and subscripts s and u denote suburban and urban areas, respectively. Subplot (e) in each row provides an overall distribution for all trip types combined. The x-axis represents the ratio of predicted to observed flows, with the vertical dashed line at 1 indicating perfect prediction. The SSUG model consistently shows peaks closer to 1 across most trip types and regions, indicating higher accuracy in predictions compared to the other models. Note that Sendai's O_u–D_u type of trips are not as predicted as accuratly as the other regions by the SSUG model.

6 Commuting Propensities

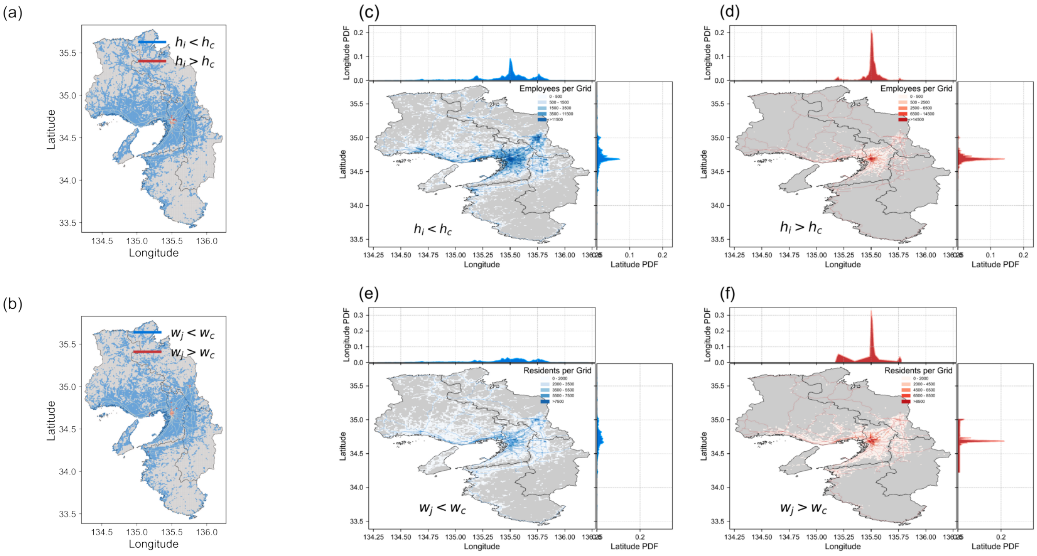

(a) Osaka

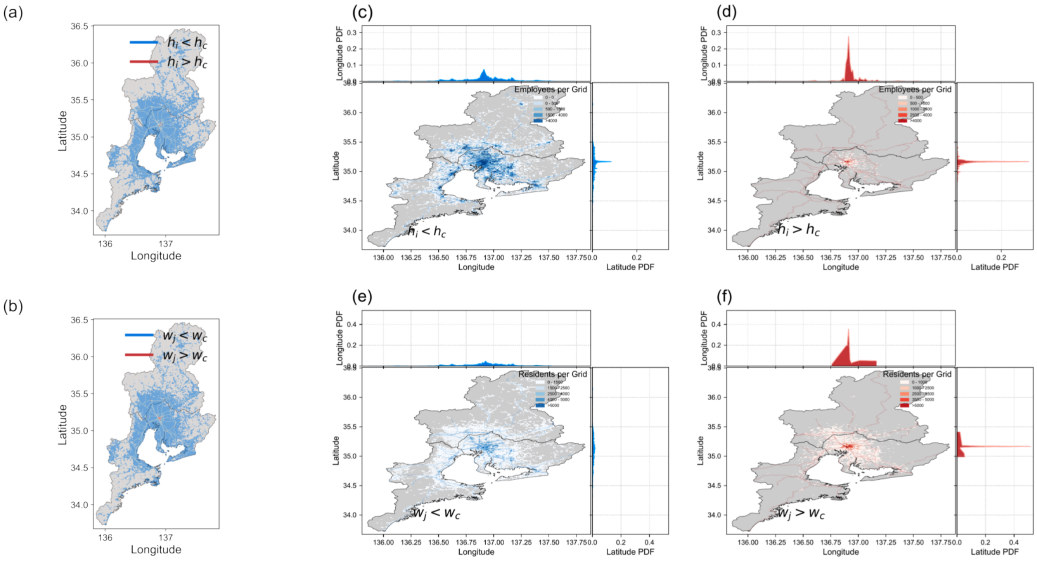

(b) Nagoya

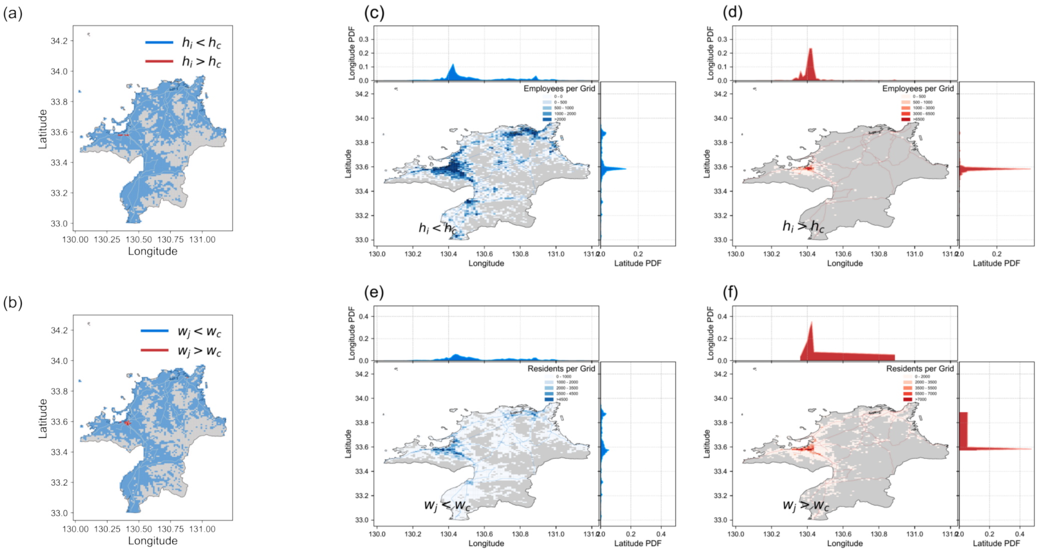

(c) Fukuoka

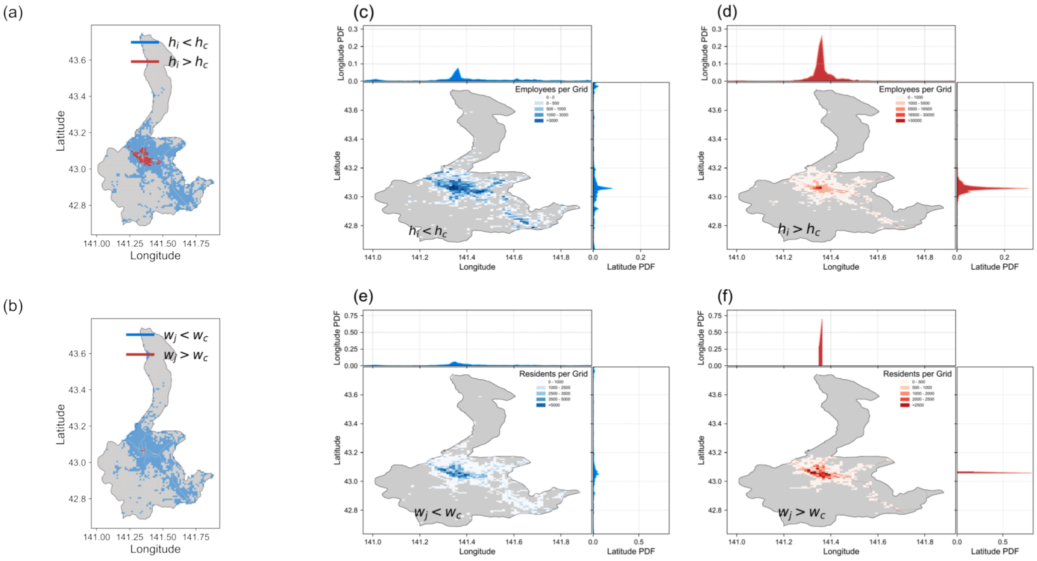

(d) Sapporo

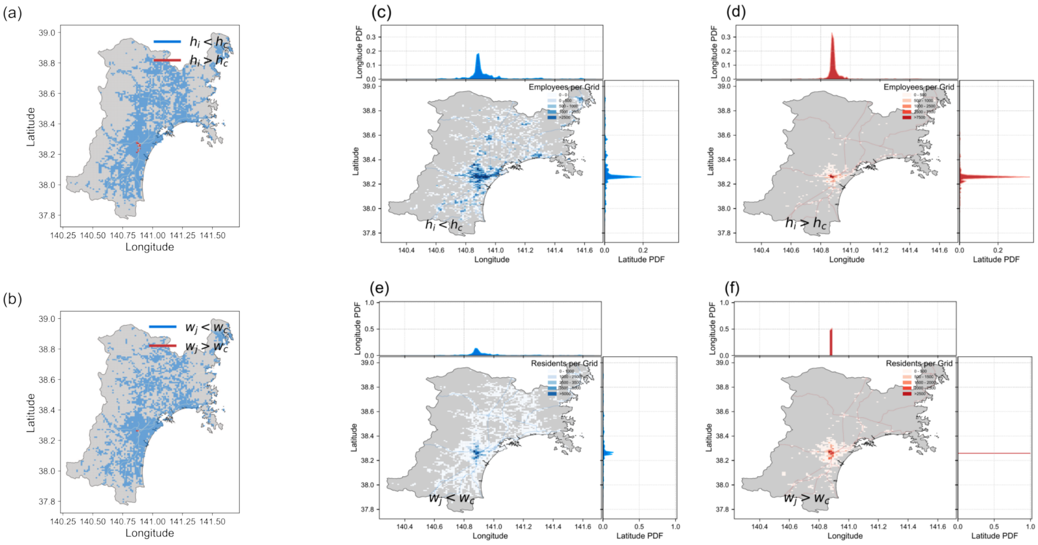

(e) Sendai

Figure S7: Spatial distribution of residential and employment populations across different metropolitan areas in Japan (Osaka, Nagoya, Fukuoka, Sapporo, and Sendai). For each region, six subplots are presented: (a) and (b) depict the spatial distribution of residential and employment populations, respectively, in 1 km^2 grid cells. Blue grids represent areas where the population (h_i or w_j) is below the threshold (h_c or w_c), while red grids indicate areas above the threshold, showing higher-density zones. (c) and (d) present the distribution of workplaces for suburban ($h_i < h_c$) and urban ($h_i > h_c$) residents, respectively. (e) and (f) show the residential distribution of suburban ($w_j < w_c$) and urban ($w_j > w_c$) employees, respectively. The main maps in each subplot display the geographical distribution, while the side panels show the Probability Density Function (PDF) of population density along longitude and latitude. This figure highlights the same urban-attracting commuting pattern across different metropolitan areas, revealing the concentrated commuting propensities for both urban residents and emplyees. Base map reprinted from [2] under a CC BY license, with permission from Ministry of Land, Infrastructure, Transport and Tourism, original copyright 2008.

7 Region Analysis

Table 1: Calibration Results of the SSUG Model for Six Japanese Metropolitan Areas

City	h_c	w_c	α_1	α_2	β_1	β_2	δ	K	CPC
**Tokyo**	12143	13445	0.09	1.9	0.01	1.3	0.98	48.73	0.26
**Osaka**	9463	14069	0.01	0.1	0.01	1.06	1.12	62.39	0.25
**Nagoya**	7963	13135	0.07	1.47	0.01	0.87	0.99	54.31	0.22
**Fukuoka**	10121	17687	0.08	2.0	0.08	1.0	1.11	52.98	0.27
**Sendai**	3210	19327	0.01	0.31	0.01	0.69	0.96	76.04	0.21
**Sapporo**	5336	40962	0.12	0.25	0.23	1.23	1.11	10.78	0.28

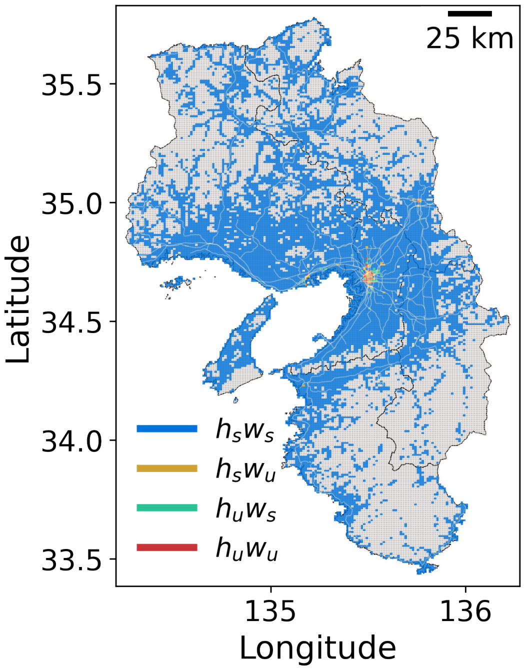

(a) Osaka

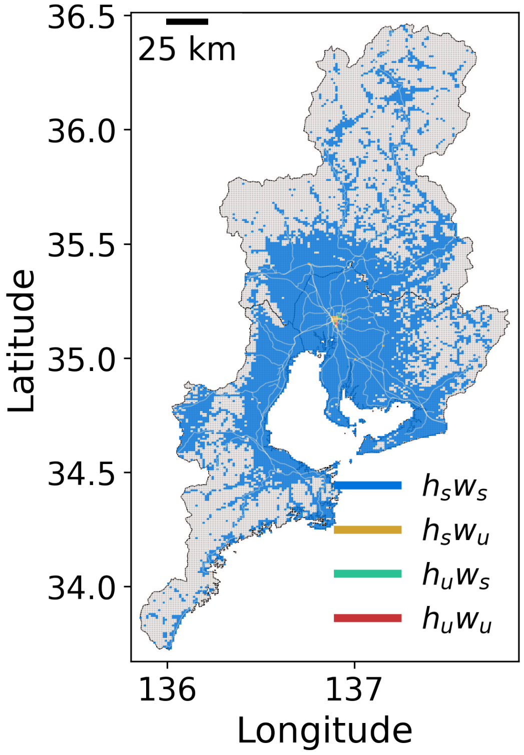

(b) Nagoya

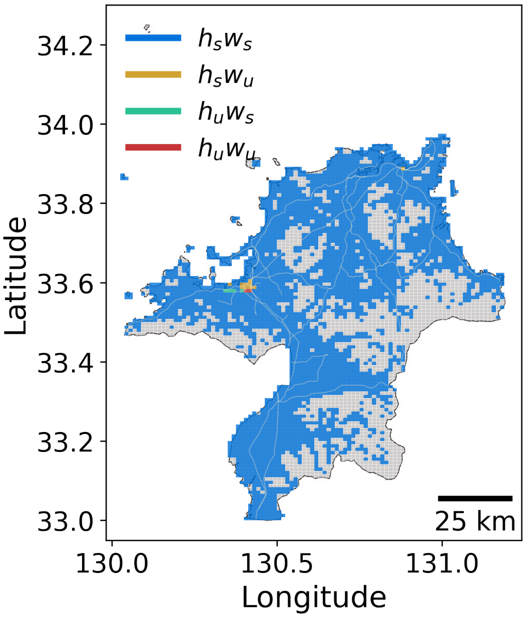

(c) Fukuoka

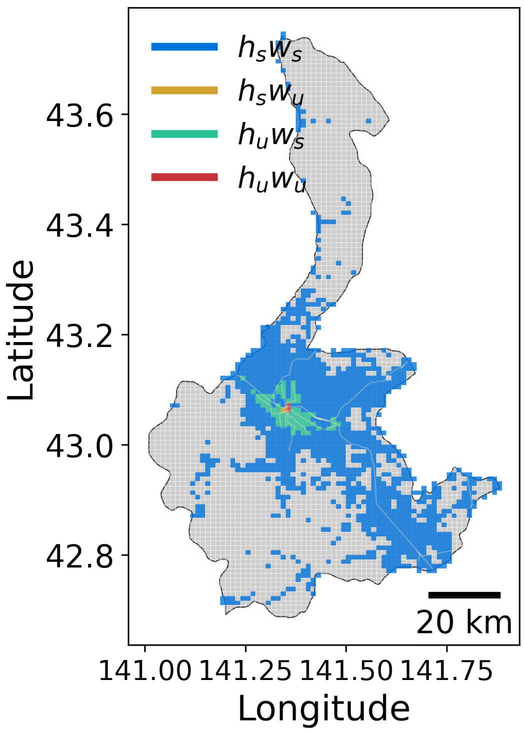

(d) Sapporo

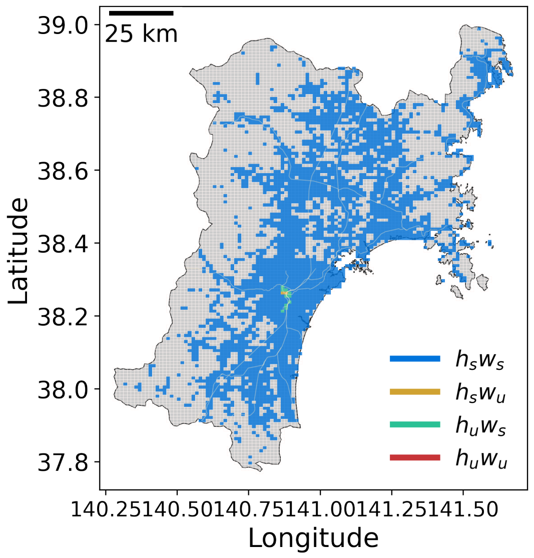

(e) Sendai

Figure S8: Spatial distribution of the four mesh types within different metropolitan areas in Japan, categorized by residential and workplace classification. The main image shows Tokyo as an example, while the smaller images represent Osaka, Nagoya, Fukuoka, Sapporo, and Sendai. The color coding represents the following categories: blue ($h_s w_s$) for suburban residential areas with suburban workplaces, yellow ($h_s w_u$) for suburban residential areas with urban workplaces, green ($h_u w_s$) for urban residential areas with suburban workplaces, and red ($h_u w_u$) for urban residential areas with urban workplaces. In all metropolitan areas, blue areas dominate the periphery, representing extensive suburban zones, while the red areas, concentrated in the center, indicate dense urban cores where both residents and employees are located. The yellow and green areas represent transitional zones between urban and suburban areas, highlighting the complex spatial structure of these metropolitan regions. This figure demonstrates how the SSUG model captures the varying urban-suburban dynamics across different Japanese metropolitan areas. Note that there is no $h_u w_u$ in Sendai region. Base map reprinted from [2] under a CC BY license, with permission from Ministry of Land, Infrastructure, Transport and Tourism, original copyright 2008.

8 Robustness Analysis

We tested whether the solution could be obtained by simple OLS. To do so, we reformatted the complete SSUG Model into logarithmic form (Eq.1), using dummy parameters to reflect the thresholds h_c and w_c. Table 2 presents a comparison between the BO results (shown in Manuscript Table 2) and OLS estimation of the linearized models:

$$\begin{aligned}
&\text{Model 1: } \log f_{ij} = \log K + \alpha_1 \log h + \beta_1 \log w - \delta \log t \\
&\text{Model 2: } \log f_{ij} = \log K + \alpha_1 \log h + \beta_2 \log w - \delta \log t - \beta_2 \log w_c + \beta_1 \log w_c \\
&\text{Model 3: } \log f_{ij} = \log K + \alpha_2 \log h + \beta_1 \log w - \delta \log t - \alpha_2 \log h_c + \alpha_1 \log h_c \\
&\text{Model 4: } \log f_{ij} = \log K + \alpha_2 \log h + \beta_2 \log w - \delta \log t - \alpha_2 \log h_c + \alpha_1 \log h_c - \beta_2 \log w_c + \beta_1 \log w_c
\end{aligned} \quad (1)$$

Parameter	h_c	w_c	α_1	α_2	β_1	β_2	δ
OLS	12560	11752	0.13	2.01	0.03	1.31	-1.02
BO	12143	11445	0.09	1.9	0.01	1.3	-0.98

Table 2: Parameter estimates using OLS and Bayesian Optimization (BO)

The similar results between OLS and BO methods support the robustness of our parameter estimates. Additionally, we conducted further robustness tests by randomly selecting 90% of the 1km grids multiple times (Table 3). The results show consistent parameter estimates across these subsamples, further confirming the stability of our model.

Parameter	h_c	w_c	α_1	α_2	β_1	β_2	δ
Run 1	11933	10933	0.10	2.10	0.03	1.31	-0.98
Run 2	12443	11443	0.08	1.74	0.03	1.31	-0.98
Run 3	12512	11512	0.11	2.20	0.03	1.31	-0.98
Run 4	12512	11512	0.11	1.72	0.03	1.31	-0.98
Run 5	12512	11512	0.11	1.72	0.02	1.31	-0.98
Run 6	12069	11069	0.10	1.74	0.03	1.31	-0.98
Run 7	12508	11508	0.09	1.87	0.03	1.31	-0.98
Run 8	12506	11506	0.10	1.76	0.02	1.31	-0.98
Run 9	12512	11512	0.11	1.84	0.03	1.31	-0.98
Run 10	12512	11512	0.10	2.02	0.01	1.31	-0.98
Run 11	12512	11512	0.11	1.72	0.03	1.31	-0.98
Run 12	12512	11512	0.10	1.72	0.03	1.31	-0.98

Table 3: Robustness tests: Randomly selecting 90% of the study units - 1km grids, take tokyo metropolitan area as an example

Also, we conducted a robustness check by recalibrating our model with the mean and median commuting times. The results, shown in Fig. 9, confirm that the model's structure is robust, as it produces reasonable predictions with all three metrics.

This outcome is consistent with the statistical properties of the commuting data. The mean is highly sensitive to outliers—unusually long trips caused by traffic or detours—which explains its lower accuracy. Mathematically, given that the commuting times follow an exponential distribution (as shown in Supplement S3), the probability mass is heavily concentrated on shorter trips. Both the median and minimum are robust measures that capture this central tendency effectively. The minimum time, in particular, likely represents the ideal, uninfluenced travel cost between two points, providing the most stable and accurate deterrence factor for the model.

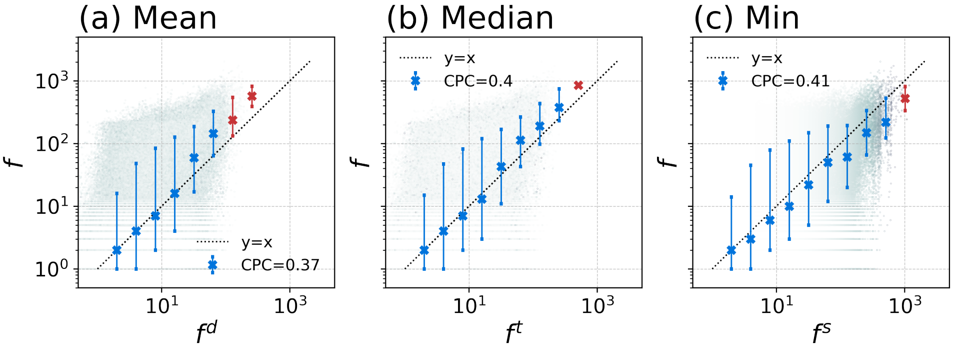

Figure S9: Robustness test results comparing model performance using minimum, mean, and median commuting times

9 Data bias

We acknowledge that our results depend on the quality of the GPS dataset. To assess potential bias, we validated our population estimates against official statistics from the Ministry of Land, Infrastructure, Transport and Tourism of Japan [3]. As shown in Figure 10, our GPS-derived population estimates are approximately half of the official population counts, with the relationship being consistently proportional across different areas. This consistent ratio suggests no obvious spatial bias in our GPS sample, as the underrepresentation appears uniform rather than concentrated in specific demographic or geographic regions.

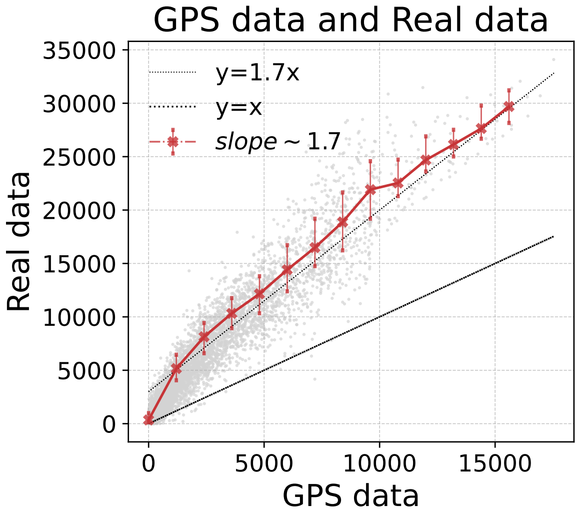

Figure S10: Comparison between GPS-derived commuting residents and actual residential population. The grey dots show individual data points, while the red line with error bars represents binned averages with a slope of approximately 1.7. Two reference lines are shown: y = 1.7x (upper dotted line) and y = x (lower dotted line).

10 CES form of the gravity model

We tested the Constant Elasticity of Substitution (CES) formulation [1] to assess potential improvements over the traditional log-log gravity model.

The SSUG model proposed in this paper uses a log-log gravity format:

$$f_{ij}^{(k)} = K \cdot \frac{h_i^{\alpha_k} w_j^{\beta_k}}{t_{ij}^{\delta_k}}, \tag{2}$$

where $k \in \{1, 2, 3, 4\}$ represents four urban categories based on high/low residential and workplace population densities, h_i is the population at origin i, w_j is the employment at destination j, t_{ij} is the commuting cost between i and j, $\alpha_k, \beta_k, \delta_k$ are elasticity parameters, and K is a proportionality constant.

We compared this with the more flexible CES form:

$$f_{ij}^{(k)} = K \cdot \left(\lambda_k h_i^{\rho_k} + (1 - \lambda_k) w_j^{\rho_k}\right)^{\frac{\sigma_k}{\rho_k}} \cdot t_{ij}^{-\delta_k}, \tag{3}$$

where λ_k is the relative weight of h_i, ρ_k controls the elasticity of substitution between h_i and w_j, σ_k is the scale elasticity parameter, and δ_k is the travel cost decay parameter.

- When $\rho_k \to -\infty$ (very negative):
  - No substitution is possible $\to$ If there aren't enough jobs (w), people cannot commute elsewhere
  - Example: A factory town where each worker must work in the same place, or they have no job
  - Mathematical Equivalent: Leontief function (fixed proportions)
- When $\rho_k = 1$ (Cobb-Douglas case):
  - Some substitution is possible, but there's a balance $\to$ increasing h slightly compensates for lower w, and vice versa
  - Example: A mixed-use urban area where jobs and residences are somewhat interchangeable
- When $\rho_k = 0$ (log-log format, the SSUG model applied):
  - Perfect substitutes $\to$ A lack of local jobs means people will fully commute elsewhere
  - Example: Suburban regions where people freely substitute between multiple employment centers

We estimated the parameters of the CES format's model using the same data without dividing the ODs by h_c and w_c, yielding the following results, with cities arranged in descending order by population size:

City	K	λ	ρ	σ	δ
Tokyo	1.18	0.37	-0.25	0.58	0.58
Osaka	0.86	0.33	-0.24	0.62	0.56
Nagoya	1.07	0.24	-0.48	0.60	0.48
Fukuoka	0.17	0.24	-0.67	0.85	0.53
Sapporo	1.29	0.08	-0.81	0.60	0.51
Sendai	0.24	0.04	-1.64	0.80	0.47

Table 4: CES model parameters for the same six Japanese cities, ordered by population size.

We found significant variation across metropolitan areas. As the parameter indicating ubstitutability ρ decreasing as the city scale decreases.

Our analysis reveals interesting patterns in how commuting behavior varies with city size. While the values of ρ across cities are close to 0, suggesting that the original SSUG model's log-log formulation is reasonable, the systematic variation in ρ provides valuable insights into urban structure. Larger cities like Tokyo and Osaka show moderate complementarity between residential and workplace locations ($\rho \approx -0.25$), while smaller cities exhibit stronger fixed relationships (e.g., Sendai with $\rho = -1.64$).

The CES analysis provides additional mathematical support for these observed patterns, particularly in how larger cities offer more workplace choice flexibility while smaller cities show more constrained commuting patterns.

11 Clarification of Constraint 2 to (Eq. 4)

At the threshold values ($h_i = h_c$, $w_j = w_c$), all four models must predict identical flows to ensure continuity.

When:

City	Commuting Behavior	Interpretation of ρ
Tokyo	Many job centers, people can choose between different workplaces	$\rho = -0.25 \rightarrow$ Moderate complementarity between h and w, some substitutability
Osaka	Similar to Tokyo but slightly more centralized	$\rho = -0.24 \rightarrow$ Similar dynamics, slightly more workplace concentration
Nagoya	More centralized, lower employment flexibility	$\rho = -0.48 \rightarrow$ More complementarity, job-housing dependency is stronger
Fukuoka	Smaller, more concentrated job clusters	$\rho = -0.67 \rightarrow$ Workers more dependent on a few employment centers
Sapporo	Larger geographic spread, limited workplace flexibility	$\rho = -0.81 \rightarrow$ Commuting constrained by long distances
Sendai	Very small, employment almost fully determines commuting	$\rho = -1.64 \rightarrow$ Almost no substitutability, fixed employment-residence relationships

Table 5: Commuting behavior patterns and interpretation of ρ across different Japanese cities

1. **Model$_1$**

$$K_1 \frac{h_c^{\alpha_1} w_c^{\beta_1}}{t_{ij}^{\delta}} = f_{ij} \tag{4}$$

$$\Rightarrow K_1 = t_{ij}^{-\delta} \tag{5}$$

2. **Model$_1$ = Model$_2$:**

$$K_1 \frac{h_c^{\alpha_1} w_c^{\beta_1}}{t_{ij}^{\delta}} = K_2 \frac{h_c^{\alpha_1} w_c^{\beta_2}}{t_{ij}^{\delta}} \tag{6}$$

$$\Rightarrow K_2 = K_1 \frac{w_c^{\beta_1}}{w_c^{\beta_2}} \tag{7}$$

3. **Model$_1$ = Model$_3$:**

$$K_1 \frac{h_c^{\alpha_1} w_c^{\beta_1}}{t_{ij}^{\delta}} = K_3 \frac{h_c^{\alpha_2} w_c^{\beta_1}}{t_{ij}^{\delta}} \tag{8}$$

$$\Rightarrow K_3 = K_1 \frac{h_c^{\alpha_1}}{h_c^{\alpha_2}} \tag{9}$$

4. **Model$_1$ = Model$_4$:**

$$K_1 \frac{h_c^{\alpha_1} w_c^{\beta_1}}{t_{ij}^{\delta}} = K_4 \frac{h_c^{\alpha_2} w_c^{\beta_2}}{t_{ij}^{\delta}} \tag{10}$$

$$\Rightarrow K_4 = K_1 \frac{h_c^{\alpha_1} w_c^{\beta_1}}{h_c^{\alpha_2} w_c^{\beta_2}} \tag{11}$$

12 OD flow and commuting driver

Fig.S11(a) displays the raw Origin-Destination (OD) commuter flows across the Tokyo metropolitan area, while Fig.11(b) shows our four-part functional grid classification. A visual comparison reveals that the most intense commuter flows form a clear structure, connecting the dense residential areas (green, $h_u w_s$) and outer suburbs (blue, $h_s w_s$) with the central urban cores (red, $h_u w_u$) and the suburban employment hubs (yellow, $h_s w_u$). The major traffic volumes are thus generated precisely by the spatial separation of these functionally different zones, validating our classification approach.

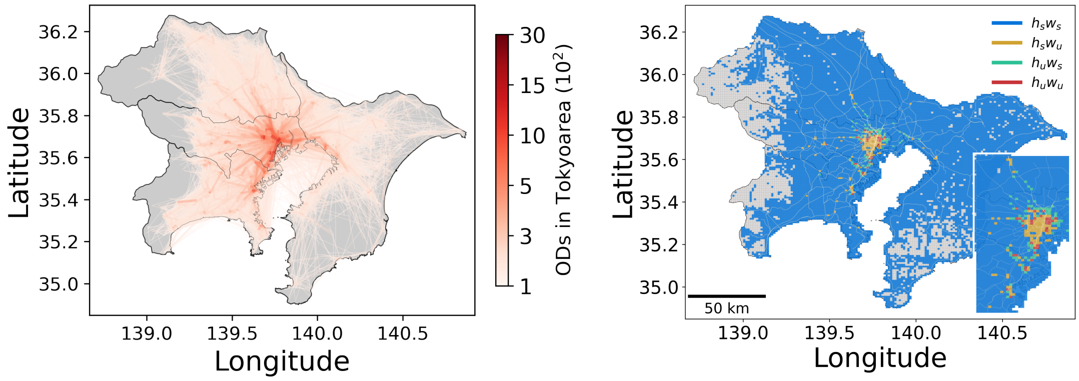

(a) (b)

Figure S11: **Commuter Flows and Functional Grid Classification in the Tokyo Metropolitan Area.** (a) Volume of daily Origin-Destination (OD) commuter flows, where red wide lines indicate a higher number of commuters (in thousands). (b) Spatial distribution of the four functional grid types based on residential (h) and workplace (w) population densities. The comparison illustrates that the largest commuter flows in (a) are generated by the functional mismatch between different grid types in (b), particularly linking residential-dominant areas ($h_u w_s$ and $h_s w_s$) with employment-dominant hubs ($h_u w_u$ and $h_s w_u$).

13 Objective Function Values

The final values of the objective function (defined in the manuscript's Eq. (6)) for each of our tested models.

**Model**	**Objective Function Value**
SSUG Model	-0.1290
Time-based Gravity Model	-7.81×10^{-12}
Distance-based Gravity Model	-1.23×10^{-14}

Table 6

As shown in Table 6, since we optimized the model by minimizing the objective function, the lowest (most negative) value of -0.1290 corresponds to the best performance. Therefore, according to the defined optimization goal, the SSUG model achieved the best result, followed by the Time-based Gravity model, with the Distance-based Gravity model performing least effectively.

14 Clearer subplots for Figure 4

References

[1] Arrow, K. J., Chenery, H. B., Minhas, B. S., and Solow, R. M. Capital-labor substitution and economic efficiency. *The Review of Economics and Statistics 43*, 3 (1961), 225–250.

[2] Ministry of Land, Infrastructure, Transport and Tourism. National Land Numerical Information - Administrative Zones Data, 2025. Accessed: February 26, 2025.

[3] Statistics Bureau of Japan. Statistics bureau home page, 2024. Accessed: February 22, 2024.

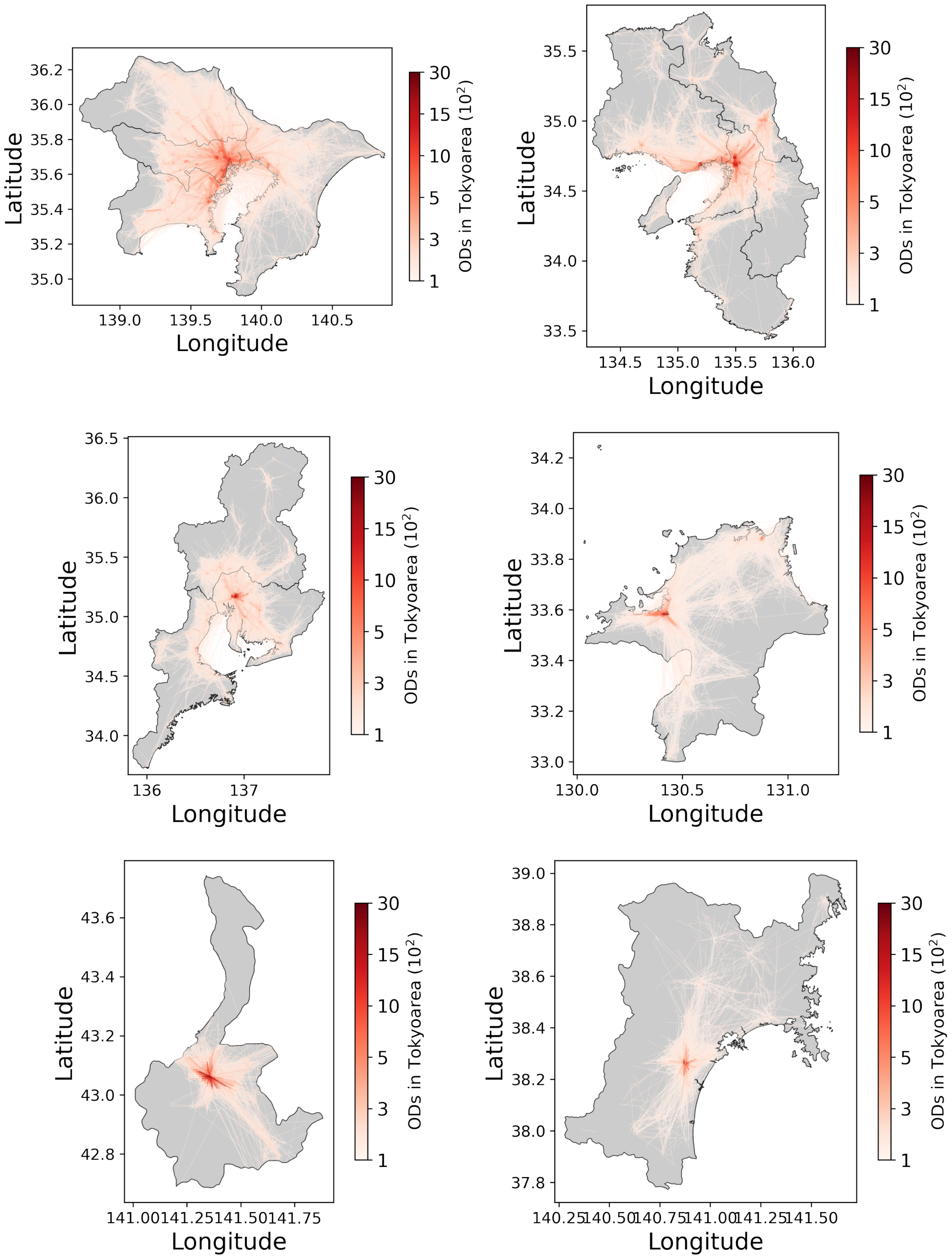

Figure S12: **Commuter Flows for all the study areas.**

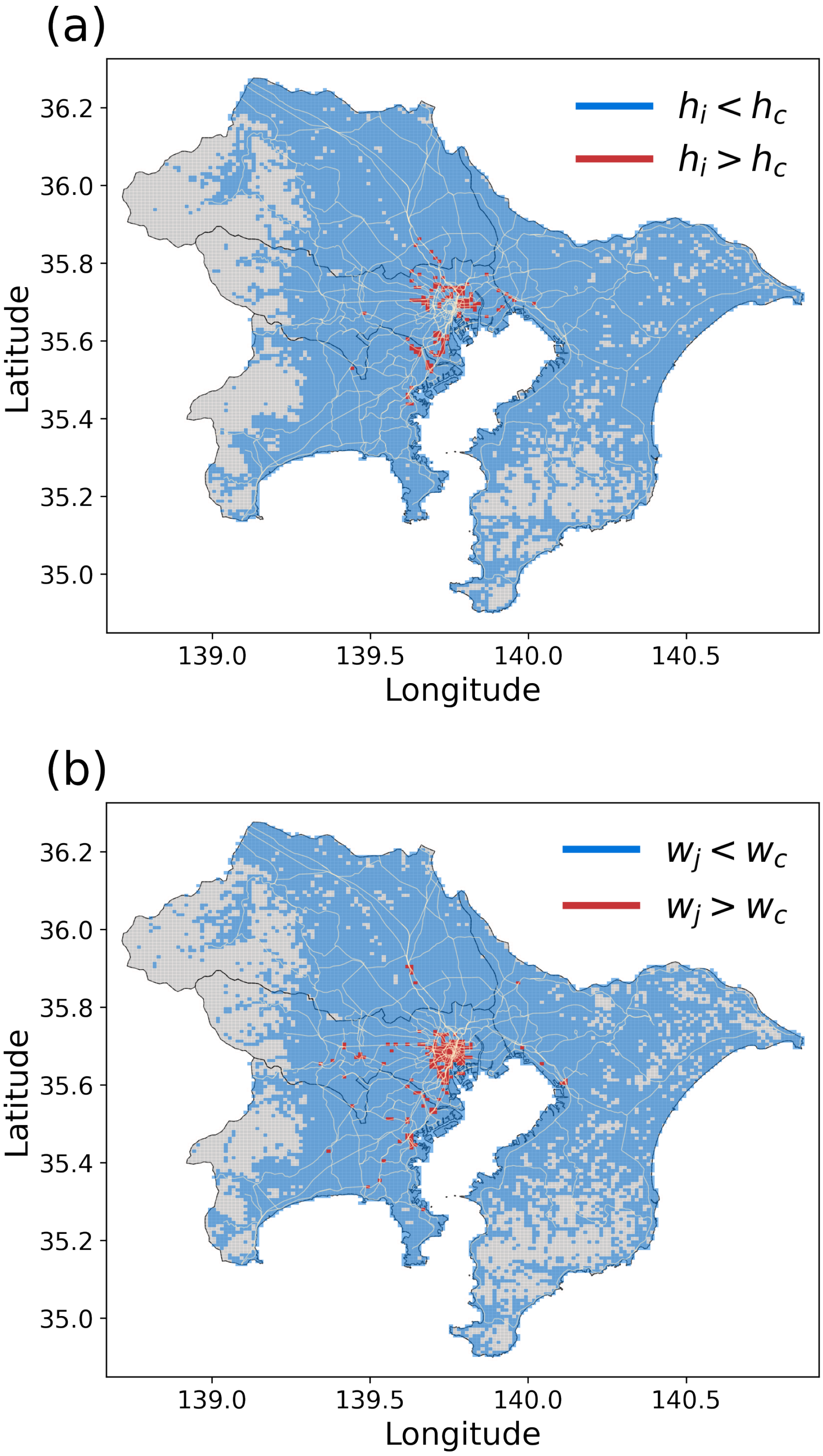

(a)
$h_i < h_c$
$h_i > h_c$
36.2
36.0
35.8
35.6
35.4
35.2
35.0
Latitude
139.0
139.5
140.0
140.5
Longitude
(b)
$w_j < w_c$
$w_j > w_c$
36.2
36.0
35.8
35.6
35.4
35.2
35.0
Latitude
139.0
139.5
140.0
140.5
Longitude

Figure S13

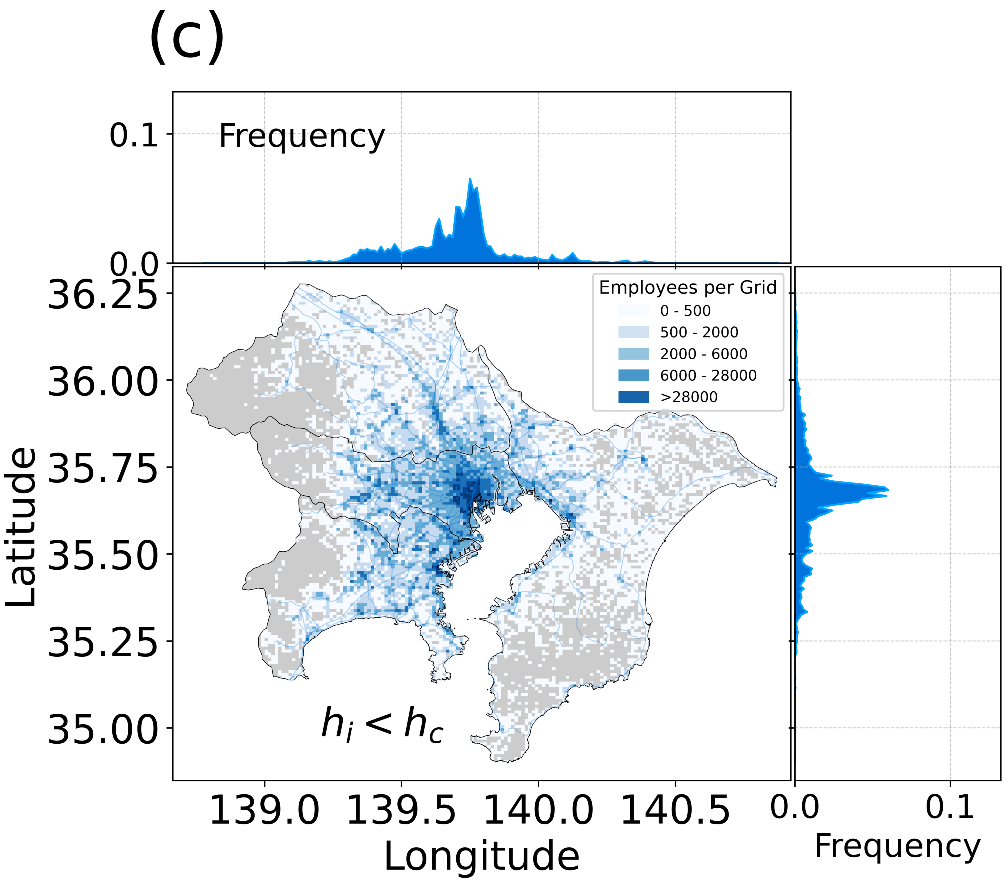
(c)
Frequency
0.1
0.0
Employees per Grid
0 - 500
500 - 2000
2000 - 6000
6000 - 28000
>28000
36.25
36.00
35.75
35.50
35.25
35.00
Latitude
$h_i < h_c$
139.0
139.5
140.0
140.5
Longitude
0.0
0.1
Frequency

Figure S14

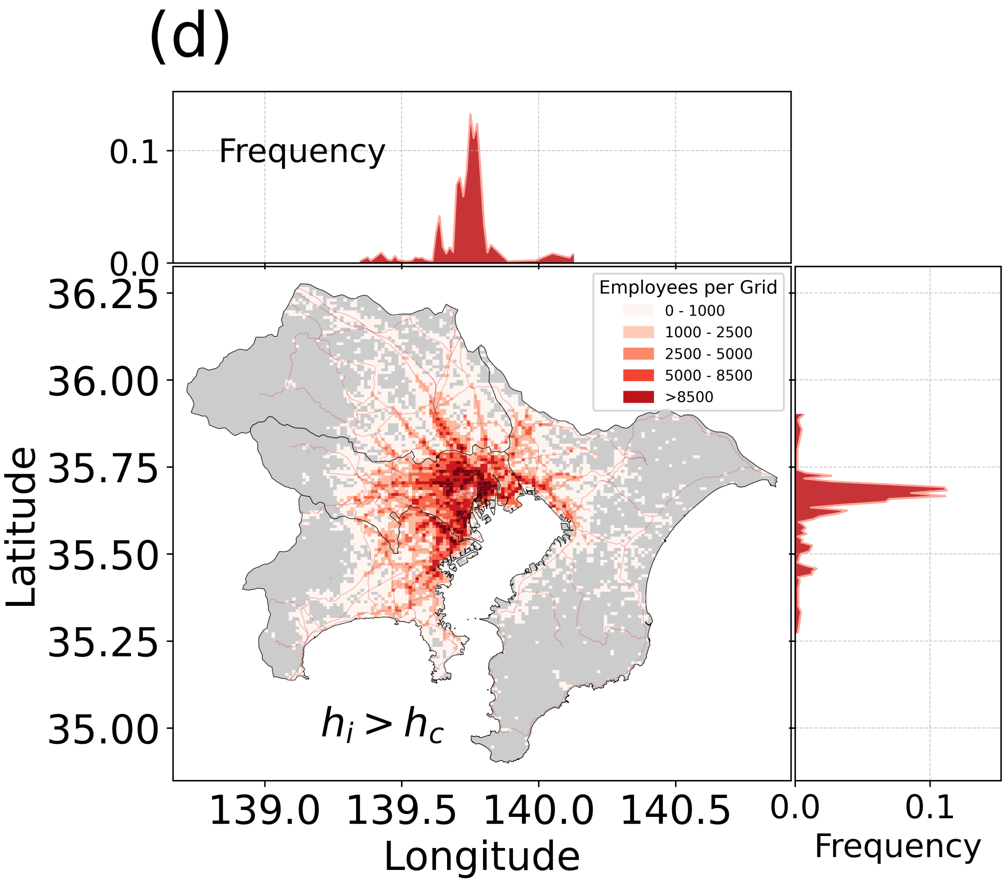
(d)
0.1
0.0
Frequency
36.25
36.00
35.75
35.50
35.25
35.00
Latitude
Employees per Grid
0 - 1000
1000 - 2500
2500 - 5000
5000 - 8500
>8500
$h_i > h_c$
139.0
139.5
140.0
140.5
Longitude
0.0
0.1
Frequency

Figure S15

(e)

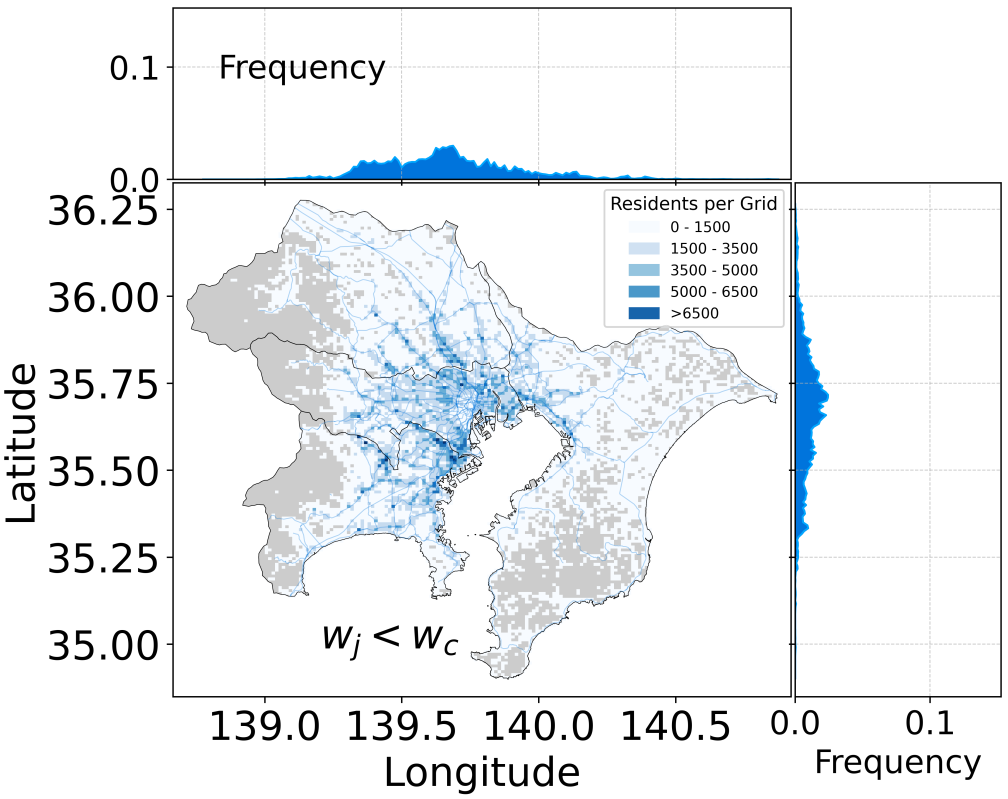

Frequency
0.1
0.0
Residents per Grid
0 - 1500
1500 - 3500
3500 - 5000
5000 - 6500
>6500
36.25
36.00
35.75
35.50
35.25
35.00
Latitude
$w_j < w_c$
139.0
139.5
140.0
140.5
Longitude
0.0
0.1
Frequency

Figure S16

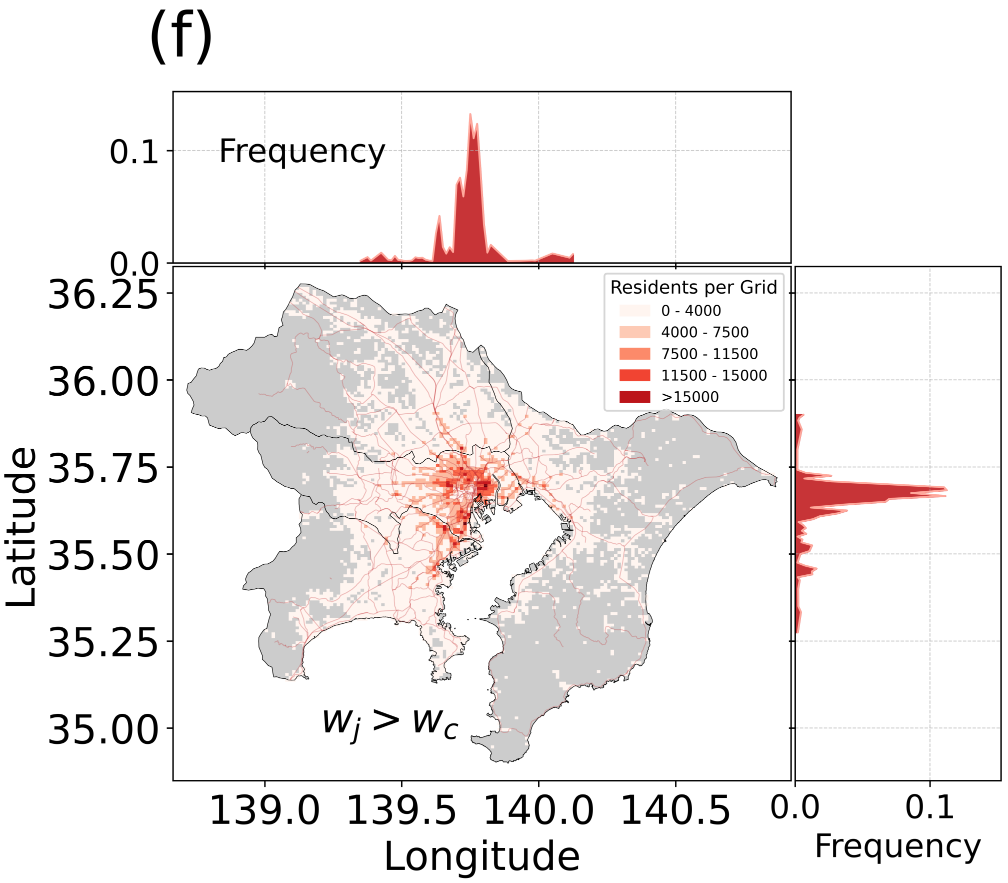
(f)
Frequency
0.1
0.0
36.25
36.00
35.75
35.50
35.25
35.00
Latitude
Residents per Grid
0 - 4000
4000 - 7500
7500 - 11500
11500 - 15000
>15000
$w_j > w_c$
139.0
139.5
140.0
140.5
Longitude
0.0
0.1
Frequency

Figure S17
