# Supplementary material for: Enhancing the gravity model for commuters: Time-and-spatial-structure-based improvements in Japan’s metropolitan areas
Source: PLoS One. 2025 Aug 12;20(8):e0329603. doi: 10.1371/journal.pone.0329603 (PMC12342275; doi:10.1371/journal.pone.0329603)
Supplement: Attachment — Submitted filename: comments.pdf [file pone.0329603.s002.pdf]

1. Motivation and Contribution to the Extant Literature

While the authors elaborate on the incentives and research gap motivating this study, the real contribution remains somewhat limited. The primary novelty lies in employing smartphone data to analyze commuting patterns across six metropolitan areas in Japan, building on the SSGU model. We recommend the authors explicitly clarify the fundamental contribution of their study in advancing the understanding and identification of urban commuting flows.

1.1 . In Section 0.3, there is no citation to support the origin and evolution of the SSGU model. In fact, SSGU has evolved in multiple directions, such as the developments discussed by He et al. (2024). Including this context would strengthen the theoretical foundation of the manuscript.

1.2 Additionally, the presentation of figures in the manuscript is suboptimal. All figures are located at the end of the document, presented without titles, and suffer from poor image quality. However, in the supplementary file, the figures (S#) are in good condition. This inconsistency impedes the reading experience and should be rectified to ensure clarity and readability throughout the paper.

1.3 Lastly, a potential weakness of the SSGU model lies in its inability to incorporate multimodal transportation options (e.g., cycling, public transit). This limitation may lead to inaccurate estimates of the influence of accessibility on commuting flows and should be acknowledged more explicitly.

2. Research Design and Methodology

2.1 The authors emphasize that urban systems are dynamic, characterized by evolving infrastructure, demographic shifts, and economic trends. However, static SSGU models may fail to account for these dynamics, thereby reducing their predictive validity. While the manuscript notes that results in 2021 are consistent with those of 2020, it would be more informative to present the results alongside an analysis of spatial-temporal changes.

2.2 The SSGU model relies heavily on parameter estimation in Equation (5). Parameters α,β, and δ are estimated using simple OLS, but robustness analyses should be included to enhance the credibility of the results. These analyses are especially important in comparison to other gravity models in Table 2. Moreover, segregation factors may be endogenously related to commuting flows, leading to potential biases in estimation. For example, high-income groups may choose to live closer to employment centers, complicating causal inferences. Addressing these issues would significantly strengthen the study's methodology.

2.3 Regarding data sources, the manuscript uses smartphone data, which is partially open to public universities. It would be helpful for the authors to clarify the differences between the Agoop Data and NTT-DOCOMO Data to enhance transparency and reproducibility.

3. Minor Points

3.1 On page 6, line 219, the parameter θ should be δ.

3.2 Figure titles in the manuscript are mismatched, making it difficult to associate figures with the appropriate content. This should be corrected.

He, Mingyi and Lyu, Mingdong and Sun, Bingrong and Belyi, Alexander and Sobolevsky, Stanislav and Garikapati, Venu, Gravattn: A Spatially Transferable Gravity Model for Trip Distribution. Available at SSRN: https://ssrn.com/abstract=5013948 or http://dx.doi.org/10.2139/ssrn.5013948

Chiawei Hsu, Chao Fan, Ali Mostafavi. Limitations of gravity models in predicting fine-scale spatial-temporal urban mobility networks.
