# Supplementary material for: Enhancing the gravity model for commuters: Time-and-spatial-structure-based improvements in Japan’s metropolitan areas
Source: PLoS One. 2025 Aug 12;20(8):e0329603. doi: 10.1371/journal.pone.0329603 (PMC12342275; doi:10.1371/journal.pone.0329603)
Supplement: Attachment — Submitted filename: report.pdf [file pone.0329603.s004.pdf]

Referee Report on "Enhancing the Gravity Model for Commuters: Time-and-Spatial-Structure-Based Improvements in Japan's Metropolitan Areas" by Yixuan Y Zheng, Yohei Shida, Hideki Takayasu, and Misako Takayasu.

Summary

This manuscript introduces the Spatially Segregated Urban Gravity (SSUG) model, which aims to improve the prediction of metropolitan commuting flows by explicitly accounting for spatial and temporal heterogeneity. The model differentiates between urban and suburban commuting dynamics and applies a Bayesian optimization framework to calibrate model parameters using high-resolution GPS data. The empirical application to six Japanese metropolitan areas demonstrates that the SSUG model outperforms traditional gravity models in predicting commuting flows.

General Assessment

The manuscript tackles a pertinent and timely topic in urban mobility modeling, offering a data-driven approach to improve commuting flow prediction. The use of GPS data and the spatially-segregated framework are particularly compelling. Notably, the SSUG model provides a more balanced prediction compared to traditional gravity models, with only slight overestimations for extremely large flows and more accurate predictions for sparse flows and overall commuting patterns. Moreover, the SSUG model serves as a practical tool for identifying city segregation and offers a quantitative approach to assessing urbanization through metrics like the Densely Inhabited District (DID) index.

However, several technical aspects and interpretations require clarification and, in some cases, additional analyses. My detailed comments are as follows:

Comments

1. **Clarification of Model Constraints (Eq. 3)**: The manuscript introduces two constraints in Eq. (3) to ensure continuity across models. However, the specific rationale for Constraint 1 (when $h_i = 1$ and $w_j = 1$, $f_{ij} = 1$) is unclear. Could the authors provide a more intuitive interpretation or justification for this condition?

2. **Interpretation of Grid Classifications ($h_s w_u$ and $h_u w_s$)**: The terms $h_s w_u$ and $h_u w_s$ appear to denote situations where a location is suburban as a residential area but urban as a workplace. This dual role seems somewhat ambiguous in practice. Could the authors elaborate on the real-world significance of such mixed-type grids, and whether this classification is robust across metropolitan areas? It would be good if the authors can discuss specific examples obtained in the results to illustrate these mixed-type grids and their practical implications.

3. **Robustness of Commuting Time Metrics (Eq. 5)**: While the authors use the minimum commuting time among observed trips, I suggest conducting a robustness check using alternative measures (e.g., average, median commuting times) to verify that the choice of metric does not unduly affect the model's performance. Additionally,

it would be valuable to discuss why commuting times can vary for the same OD pair, especially since the data indicate actual variability in these times.

4. **Statistical Validity of F-Test Application (Eq. 10)**: The authors use an F-test to assess the significance of introducing segmentation in the gravity model. However, Eq. (10) assumes that parameters are estimated via OLS under normally distributed errors, whereas the manuscript indicates that parameters are estimated using Bayesian optimization (BO). Could the authors clarify the validity of applying the F-test in this context? Additional explanation in Appendix A5.2 is warranted.

5. **CPC Evaluation Metric**: The manuscript currently uses the CPC index to assess model fit. However, since the SSUG model is calibrated via Bayesian optimization (BO), it may be simpler and more consistent to use the value of the BO objective function directly as the evaluation metric, rather than introducing an additional index. This approach would ensure coherence between the evaluation measure and the model calibration process.

Conclusion

This manuscript makes an important contribution to commuting flow modeling by incorporating spatial segregation and time-based decay factors. Addressing the above concerns will strengthen the manuscript's methodological rigor and practical relevance.
