# Supplementary material for: Enhancing the gravity model for commuters: Time-and-spatial-structure-based improvements in Japan’s metropolitan areas
Source: PLoS One. 2025 Aug 12;20(8):e0329603. doi: 10.1371/journal.pone.0329603 (PMC12342275; doi:10.1371/journal.pone.0329603)
Supplement: Attachment — Submitted filename: Response_to_Reviewers.pdf [file pone.0329603.s005.pdf]

Response to Editor and Reviewers

Dear Editor and Reviewers,

We sincerely thank the editor and all reviewers for their invaluable feedback that has significantly strengthened our manuscript.

To the Editor: We are deeply grateful for your time, patience, and commitment throughout this review process. Your willingness to continue working with our manuscript, particularly given that it had been reviewed previously, means a great deal to us and demonstrates exceptional editorial dedication. Your careful coordination of the review process have been instrumental in bringing this work to its current improved state.

To Reviewer #3: Your constructive suggestions have greatly enhanced the readability and applicability of our paper. Your guidance on clarifying methodology assumptions and limitations has been particularly beneficial, providing clear structural direction for our study. Your emphasis on making assumptions and their corresponding limitations explicit first has improved our research framework significantly. Additionally, your focus on transferability highlighted the key value of our study for future development in this research field.

To Reviewer #4: Your detailed theoretical suggestions have substantially enhanced the credibility of our work from a fundamental perspective. Your comprehensive comments on assumptions, constraint settings, and result comparisons were especially valuable in strengthening our methodological rigor. Most importantly, your request for clearer explanation of the $h_u w_s$ and $h_s w_u$ areas' classifications helped us better articulate this key component of our paper, which forms the core innovation of our approach.

The collective feedback from all reviewers has resulted in a much stronger, clearer, and more impactful manuscript. We believe the revisions have significantly improved both the theoretical rigor and practical applicability of our research.

Thank you again for your dedication to advancing scholarly research in this field.

Response to Reviewer 3

1. Methodology Assumptions and Limitations: "I would like to suggest a broader explanation of the assumptions of the methodology and how these assumptions limit the conclusions. Specifically, please discuss the assumption that in the model individuals always take the fastest route and its implications for the study's findings."

Response:

We appreciate the reviewer pointing out this important limitation. The primary assumption of our methodology is that commuters optimize their journey, which we model using the minimum observed travel time for each Origin-Destination pair. We have addressed this concern by expanding our discussion of methodology assumptions and their implications in the manuscript conclusion.

Content added to manuscript conclusion: line 725: A further limitation of this study lies in the assumption that commuting time for an OD pair corresponds to the minimum observed travel time. While this approach is justified by the principle of travel time optimization and helps filter out non-commuting detours, it represents a simplification of real-world behavior. The model therefore does not fully account for the variability in travel times caused by different transportation modes, route choices, and real-time congestion. This assumption may impact the model's conclusions; for example, the overestimation of flows for certain trip types, such as from suburban origins to urban destinations, could be partially explained by this limitation. In these regions, a small number of individuals with access to faster private transport may define the minimum travel time, while the majority

who rely on slower public transport are not fully represented. Future research could explore more complex deterrence functions that account for modal choice and travel time variability.

We have also conducted robustness tests using mean and median travel times (presented in Supplement Section 8) to validate that our model structure remains stable under different travel time assumptions.

We note that additional key limitations have been addressed in the manuscript conclusion, including:

- **Dataset context:** Our analysis uses data from 2021, a period with potentially atypical travel patterns, and from a source with a known demographic bias against elderly individuals.
- **Model scope:** While our model effectively uses population density and travel time to predict commuting flows, it does not include other factors that influence commuting, such as public transportation accessibility, socioeconomic variables (such as income), or the specific locations of major employers.

These biases have been thoroughly discussed in the manuscript's Conclusion section.

2. Urban Segmentation Complexity: "Is it possible to enhance the urban segmentation approach by incorporating variables beyond population density? Could other factors be considered to create a more nuanced classification system?"

Response:

We thank the reviewer for this thought-provoking question that touches on a fundamental challenge in urban modeling: the trade-off between model complexity and transferability.

We acknowledge that incorporating additional variables such as land use composition, public transportation accessibility, or economic indicators could potentially enhance our classification system and improve model performance in specific contexts. However, we deliberately adopted a parsimonious approach using only population density variables for several key reasons:

- **Aggregation consistency:** Our model operates at the 1-km grid scale, and additional variables would need to be aggregated to this same resolution. This inevitable aggregation process in this field of studies introduce additional approximation errors that may compound rather than improve model accuracy.
- **Data availability and standardization:** Population density data is consistently available across different metropolitan areas and countries with standardized definitions, whereas variables like detailed land use classifications, transportation network characteristics, or economic indicators often vary significantly in availability, resolution, and definition across different contexts.
- **Transferability and generalizability:** Each additional variable potentially reduces the model's applicability to new areas where such data may not be available or may be defined differently. Our goal is to develop a framework that can be readily applied across diverse metropolitan contexts worldwide.

We agree that exploring more complex segmentation approaches represents an important avenue for future research. potential extensions could include multi-scale analysis and dynamic classification systems that adapt to local data availability.

Our robustness tests across six diverse Japanese metropolitan areas demonstrate that the population density-based approach successfully captures fundamental urban functional patterns, suggesting that while additional complexity might offer marginal improvements, the core relationships are well-captured by our current framework.

We hope our approach strikes an appropriate balance between explanatory power and practical applicability, though we acknowledge this remains an active area for methodological development.

3. Figure and Equation Quality: "It would be beneficial to improve the quality of the illustrations and equations to make them more readable and accessible to readers."

Response:

We sincerely thank the reviewer for this valuable feedback on improving the readability and accessibility of our visual and mathematical presentations.

Equation improvements: We have revised several equations to enhance clarity and readability. For example, we amended the expression for Eq. 6 in the manuscript (line: 324) as follows:

$$\text{Objective function: } \frac{\sum_{i,j} \log\left(\frac{\hat{f_{ij}}}{f_{ij}}\right)}{\text{No. of ODs}} \tag{1}$$

By explicitly placing "No. of ODs" in the denominator and using clearer mathematical notation, the equation becomes more intuitive and easier to interpret.

Also, we should clarify how Constraint 2 leads to Equation (4). The derivation proceeds as follows:

At the threshold values ($h_i = h_c$, $w_j = w_c$), all four models must predict identical flows to ensure continuity.

When:

1. **Model$_1$ = Model$_2$:**

$$K_1 \frac{h_c^{\alpha_1} w_c^{\beta_1}}{t_{ij}^{\delta}} = K_2 \frac{h_c^{\alpha_1} w_c^{\beta_2}}{t_{ij}^{\delta}} \tag{2}$$

$$\Rightarrow K_2 = K_1 \frac{w_c^{\beta_1}}{w_c^{\beta_2}} \tag{3}$$

2. **Model$_1$ = Model$_3$:**

$$K_1 \frac{h_c^{\alpha_1} w_c^{\beta_1}}{t_{ij}^{\delta}} = K_3 \frac{h_c^{\alpha_2} w_c^{\beta_1}}{t_{ij}^{\delta}} \tag{4}$$

$$\Rightarrow K_3 = K_1 \frac{h_c^{\alpha_1}}{h_c^{\alpha_2}} \tag{5}$$

3. **Model$_1$ = Model$_4$:**

$$K_1 \frac{h_c^{\alpha_1} w_c^{\beta_1}}{t_{ij}^{\delta}} = K_4 \frac{h_c^{\alpha_2} w_c^{\beta_2}}{t_{ij}^{\delta}} \tag{6}$$

$$\Rightarrow K_4 = K_1 \frac{h_c^{\alpha_1} w_c^{\beta_1}}{h_c^{\alpha_2} w_c^{\beta_2}} \tag{7}$$

We hope this explanation improves the quality of the equations. We have now added this part to the Supplement material.

Figure improvements: We acknowledge the importance of high-quality, accessible illustrations. While PLOS ONE's submission format requires figures to be submitted separately from the manuscript, which can affect integrated readability during the review process, we redesigned Fig. 4 by presenting it as separate, larger subplots rather than a single complex multi-panel figure in the Supplement's last section, significantly improving readability. The font size has been amended correspondingly as well.

We have also reviewed all figures in the manuscript to improve their quality and accessibility. Specifically, we have ensured all figures are submitted in a high-resolution format (300 DPI).

We believe these improvements will substantially enhance the accessibility and impact of our visual presentations while maintaining the scientific rigor of our results.

4. Generalizability of Conclusions: "The conclusions should be strengthened by exploring how the findings can be transferred to territorial realities other than Japan. Please discuss the broader applicability of your methodology and results."

Response:

We thank the reviewer for raising this crucial point about the generalizability of our findings beyond Japan. We believe this methodology and results are broadly applicable to metropolitan areas worldwide for two fundamental reasons: the universality of the functional urban structures our model identifies and the transferability of our data-driven approach.

Universal Urban Functional Types

Analysis through the SSUG model reveals four distinct functional urban typologies that correspond to fundamental components of metropolitan spatial structure established in urban planning theory [1, 2]:

- **Primary urban cores ($h_u w_u$):** High-density mixed-use areas equivalent to Central Business Districts (CBDs), such as Chiyoda ward and Shinjuku ward in Tokyo
- **Residential-dominant zones ($h_u w_s$):** Dense residential areas with limited local employment, representing "bedroom communities" like Kōtō Ward
- **Employment centers ($h_s w_u$):** Suburban job concentrations or "edge cities" such as Tachikawa, providing decentralized employment opportunities
- **Low-density periphery ($h_s w_s$):** Sparsely populated areas like Okutama, characterized by minimal residential and employment density

Our cross-city analysis (Supplement Figs. S8, newly added S11, S12) reveals several key patterns: line 582: Each classification corresponds to a unique urban function with significant real-world implications for planning and mobility. The urban core ($h_u w_u$) represents the primary Central Business Districts (CBDs), such as Chiyoda ward and Shinjuku ward, characterized by a high-density mix of both corporate headquarters and residential populations; the key planning implication here is the need to manage hyper-density with high-capacity, multi-modal public transport. In contrast, areas classified as $h_u w_s$ are dense residential districts with low local employment. A clear example is Kōtō Ward, known for its large-scale apartment complexes. These zones are the epicenters of commuter demand, exporting a massive workforce daily and thus placing immense pressure on public transportation corridors.

These residential areas are complemented by employment magnets ($h_s w_u$), which are secondary urban centers or edge cities like Tachikawa in western Tokyo. These hubs are crucial for decentralization, offering major employment opportunities closer to suburban homes, which can reduce overall commute distances and relieve pressure on the urban core. Finally, the $h_s w_s$ category covers the sparsely populated periphery, such as the mountainous Okutama region. Characterized by low residential and employment densities, planning for these areas must address potential car dependency and the provision of essential local services. This spatial segregation of functions creates significant commuting patterns, as Figs. S11 and S12 demonstrate that mismatches between residential and employment populations are the main drivers of commuting flows.

Each distinct function's geographical distribution reveals a core-periphery structure with central business districts, surrounding residential zones, and secondary commercial centers. This pattern

aligns with established urban planning theory [1, 2]. The identification of these distinct functional zones is a robust finding; as shown in Supplement Fig S8, smaller cities like Fukuoka, Sapporo, and Sendai exhibit similar, albeit more isolated, core-periphery structures, highlighting universal patterns of urban density and connectivity across different metropolitan scales.

This spatial organization reflects the classic core-periphery structure of central business districts, surrounding residential zones, and secondary commercial centers described in urban planning theory [2]. While specific scales and spatial arrangements vary, the majority of major metropolitan areas contain these fundamental functional types.

Transferable Data-Driven Methodology

A key strength of our approach is its reliance solely on origin-destination commuting flow data, without dependence on Japan-specific administrative boundaries or pre-defined zoning classifications. The methodology requires only GPS-derived mobility data, which is increasingly available worldwide through mobile phone records, transportation surveys, or census data.

This data-driven foundation enables researchers in other regions to:

- Apply the same SSUG framework to discover unique functional segmentation patterns in their cities
- Identify zones that contribute most significantly to commuting flows
- Determine local-specific scaling laws and thresholds governing urban mobility
- Adapt model parameters (h_c, w_c) to reflect local transportation infrastructure and planning policies

While we expect fundamental urban structures to be universal, we anticipate that model parameters will vary across different metropolitan contexts, reflecting local variations in economic geography, transportation systems, and urban development patterns. This adaptability is a strength rather than a limitation, as it allows the model to capture context-specific characteristics while maintaining its core theoretical framework.

In manuscript, we added this part in Conclusion: line 692: The SSUG model demonstrates strong potential for generalizability beyond Japan through two fundamental characteristics. First, the functional urban zones it identifies—primary urban cores ($h_u w_u$), residential-dominant areas ($h_u w_s$), employment centers ($h_s w_u$), and low-density periphery ($h_s w_s$)—represent universal metropolitan archetypes documented across global urban systems [1, 2]. While spatial configurations vary, these functional roles in generating commuting flows are consistent across diverse metropolitan contexts.

Second, the methodology's transferability stems from its purely data-driven approach, requiring only origin-destination flow data without dependence on region-specific administrative boundaries or zoning classifications. With GPS, mobile network, or survey data increasingly available worldwide, researchers can apply this framework to identify local-specific parameters (h_c, w_c) and scaling relationships governing metropolitan mobility. The SSUG model thus provides a standardized yet adaptable framework for comparative urban analysis across diverse territorial realities.

We encourage future research to validate our findings across diverse international contexts and expect that such studies will confirm the broader applicability of our methodology while revealing interesting variations in local urban functional organization.

Response to Reviewer 4

1. Clarification of Model Constraints (Eq. 3): The manuscript introduces two constraints in Eq. (3) to ensure continuity across models. However, the specific rationale for Constraint 1 (when $h_i = 1$ and

$w_j = 1$, $f_{ij} = 1$) is unclear. Could the authors provide a more intuitive interpretation or justification for this condition?

Response:

Thank you for your valuable feedback. We appreciate the opportunity to clarify our model constraints.

Clarification of Constraint 1 ($h_i = 1, w_j = 1 \Rightarrow f_{ij} = 1$)

Constraint 1 is definitional and ensures internal consistency, because:

- **Definition** In our data, we define
$$h_i = \sum_j f_{ij}, \qquad w_j = \sum_i f_{ij}.$$
This means h_i is the total number of commuters living at i, and w_j is the total number of commuters working at j. If $h_i = 1$ and $w_j = 1$, there is exactly one commuter originating at i and exactly one commuter destined for j. By construction, that must be the same person, so $f_{ij} = 1$.
- **Scope** Since f_{ij} represents only observed commuting trips (no non-commute trips are included), there is no "extra" traveler. When both h_i and w_j equal 1, the only possible flow between i and j is that single commuter.

Thus, Constraint 1 states that when origin i has one commuter and destination j also has one commuter, the observed flow between them must be that one commuter ($f_{ij} = 1$). It provides a consistent baseline for the constant $K(K_1)$ across all four model types, ensuring continuity and interpretability of the model.

$$K_1 \frac{h_c^{\alpha_1} w_c^{\beta_1}}{t_{ij}^{\delta}} = f_{ij} \tag{8}$$
$$\Rightarrow K_1 = t_{ij}^{-\delta} \tag{9}$$

We have amended the text in line 251: Constraint 1 states that when origin i has one commuter and destination j also has one commuter, the observed flow between them must be that one commuter (($f_{ij} = 1$). It provides a consistent baseline for the constant $K(K_1)$ across all four model types, ensuring continuity and interpretability of the model. These constraints transform our initial formulations into more complex expressions with consistent behavior at boundary conditions (For clarification, please see Supplement Section 11).

Clarification of Constraint 2 to (Eq. 4)

As the reviewer noted, we should clarify how Constraint 2 leads to Equation (4). The derivation proceeds as follows:

At the threshold values ($h_i = h_c$, $w_j = w_c$), all four models must predict identical flows to ensure continuity.

When:

1. **Model$_1$ = Model$_2$:**
$$K_1 \frac{h_c^{\alpha_1} w_c^{\beta_1}}{t_{ij}^{\delta}} = K_2 \frac{h_c^{\alpha_1} w_c^{\beta_2}}{t_{ij}^{\delta}} \tag{10}$$
$$\Rightarrow K_2 = K_1 \frac{w_c^{\beta_1}}{w_c^{\beta_2}} \tag{11}$$

2. **Model_1 = Model_3:**

$$K_1 \frac{h_c^{\alpha_1} w_c^{\beta_1}}{t_{ij}^{\delta}} = K_3 \frac{h_c^{\alpha_2} w_c^{\beta_1}}{t_{ij}^{\delta}} \tag{12}$$

$$\Rightarrow K_3 = K_1 \frac{h_c^{\alpha_1}}{h_c^{\alpha_2}} \tag{13}$$

3. **Model_1 = Model_4:**

$$K_1 \frac{h_c^{\alpha_1} w_c^{\beta_1}}{t_{ij}^{\delta}} = K_4 \frac{h_c^{\alpha_2} w_c^{\beta_2}}{t_{ij}^{\delta}} \tag{14}$$

$$\Rightarrow K_4 = K_1 \frac{h_c^{\alpha_1} w_c^{\beta_1}}{h_c^{\alpha_2} w_c^{\beta_2}} \tag{15}$$

We hope this explanation clarifies the roles of both Constraint 1 and 2, and the rationale behind the SSUG model. We have now added this part to the Supplement material.

2. Interpretation of Grid Classifications ($h_s w_u$ and $h_u w_s$): The terms $h_s w_u$ and $h_u w_s$ appear to denote situations where a location is suburban as a residential area but urban as a workplace. This dual role seems somewhat ambiguous in practice. Could the authors elaborate on the real-world significance of such mixed-type grids, and whether this classification is robust across metropolitan areas? It would be good if the authors can discuss specific examples obtained in the results to illustrate these mixed-type grids and their practical implications.

Response:

We thank the reviewer for their insightful question on interpreting our mixed-type grid classifications ($h_s w_u$ and $h_u w_s$). These categories are crucial for capturing the functional complexity and polycentric nature of modern metropolitan areas.

Our model's key strength lies in its ability to diagnose the spatial mismatch between residential and employment concentrations—a mismatch we contend is the primary driver of commuting flows. This hypothesis is empirically validated by Figure 1, where the observed Origin-Destination (OD) commuter flows in Figure 1(a) align remarkably with our functional classifications in Figure 1(b) (corresponding to Figure 5 in the manuscript).

The pattern is striking: the most intense commuter flows occur between functionally mismatched areas—specifically from residential-dominant zones ($h_u w_s$) to employment-dominant zones ($h_s w_u$ and $h_u w_u$). These flows originate predominantly in areas with high residential density but limited local employment opportunities and terminate in areas with concentrated job opportunities, whether in central urban cores ($h_u w_u$) or suburban employment centers ($h_s w_u$).

Conversely, areas with balanced residential-employment ratios ($h_s w_s$) or functionally similar adjacent zones generate relatively modest commuting volumes. This spatial pattern—where major traffic volumes are generated precisely by the separation between living and working concentrations—strongly validates our classification approach and confirms that functional mismatch is indeed the fundamental driver of metropolitan commuting patterns.

Real-World Significance & Planning Implications

The model's grid classifications serve as a diagnostic tool, revealing that each grid type presents distinct urban planning challenges and opportunities:

- $h_u w_u$ **(Urban Core):** Corresponds to the primary Central Business Districts (CBDs) like **Chiyoda ward and Shinjuku ward**, characterized by a high density of both jobs and residents. The key planning implication is the need to manage hyper-density and support high-capacity, multi-modal public transport.

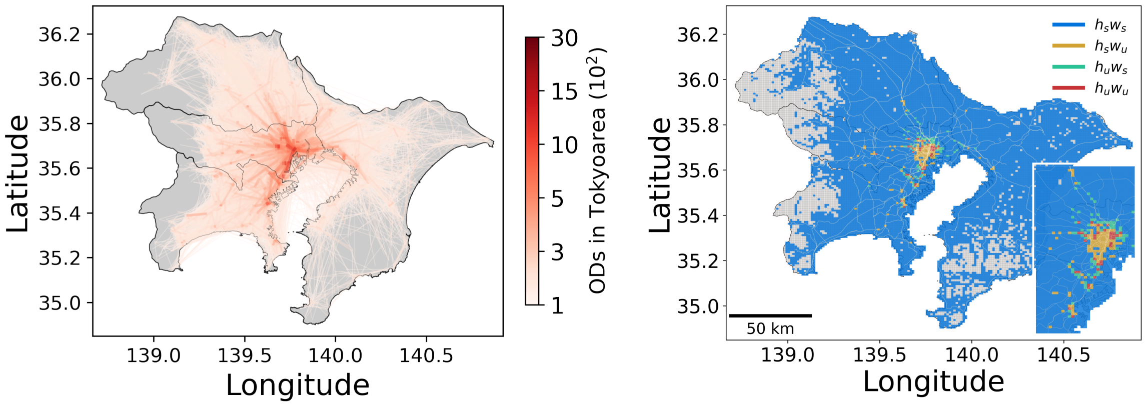

(a) (b)

Figure 1: **Commuter Flows and Functional Grid Classification in the Tokyo Metropolitan Area.** (a) Volume of daily Origin-Destination (OD) commuter flows, where red wide lines indicate a higher number of commuters (in thousands). (b) Spatial distribution of the four functional grid types based on residential (h) and workplace (w) population densities. The comparison illustrates that the largest commuter flows in (a) are generated by the functional mismatch between different grid types in (b), particularly linking residential-dominant areas ($h_u w_s$ and $h_s w_s$) with employment-dominant hubs ($h_u w_u$ and $h_s w_u$).

- $h_u w_s$ **(Residential Exporters):** Represents dense "bedroom communities" with high population but low local employment, such as the high-rise residential districts in **Kōtō Ward**. These zones are the epicenters of commuter demand, requiring efficient, high-capacity transit to export their large workforce to the urban core and secondary employment hubs daily.
- $h_s w_u$ **(Employment Magnets):** Forms secondary urban centers or "edge cities" like **Tachikawa** and **Omiya**, which act as vital employment hubs within the suburbs. For planners, these hubs are a powerful tool for decentralization, relieving pressure on the CBD and reducing overall commute distances to create a more resilient metropolitan structure.
- $h_s w_s$ **(Outer Periphery):** Covers the sparsely populated periphery, such as the mountainous **Okutama** region, with low densities of both jobs and residents. Planning for these areas must focus on providing essential local services while managing potential car dependency.

Robustness in all the metropolitan areas

The robustness of this classification is demonstrated consistently across all six Japanese metropolitan areas studied (Supplementary Figs. S8 and newly attached Fig. S11 and S12). Our data-driven approach, where thresholds h_c and w_c are optimized based on actual commuting patterns, successfully identifies functionally distinct zones in every metropolitan area analyzed, regardless of their unique urban structures. For instance, while cities like Sapporo and Sendai exhibit more centralized patterns compared to the polycentric Tokyo region, the fundamental classification logic—distinguishing residential-dominant from employment-dominant areas—remains valid and produces meaningful spatial patterns in all cases.

This classification approach provides a quantitative, non-arbitrary method for identifying functional urban types that administrative boundaries or simple population density thresholds consistently miss across diverse metropolitan contexts.

We have amended the text in line 582: Each classification corresponds to a unique urban function with significant real-world implications for planning and mobility. The urban core ($h_u w_u$) represents

the primary Central Business Districts (CBDs), such as Chiyoda ward and Shinjuku ward, characterized by a high-density mix of both corporate headquarters and residential populations; the key planning implication here is the need to manage hyper-density with high-capacity, multi-modal public transport and daytime services. In contrast, areas classified as $h_u w_s$ are dense residential districts with low local employment. A clear example is Kōtō Ward, known for its large-scale apartment complexes. These zones are the epicenters of commuter demand, exporting a massive workforce daily and thus placing immense pressure on public transportation corridors.

These residential areas are complemented by employment magnets ($h_s w_u$), which are secondary urban centers or edge cities like Tachikawa in western Tokyo. These hubs are crucial for decentralization, offering major employment opportunities closer to suburban homes, which can reduce overall commute distances and relieve pressure on the urban core. Finally, the $h_s w_s$ category covers the sparsely populated periphery, such as the mountainous Okutama region. Characterized by low residential and employment densities, planning for these areas must address potential car dependency and the provision of essential local services. This spatial segregation of functions creates significant commuting patterns, as Figs. S11 and S12 demonstrate that mismatches between residential and employment populations are the main drivers of commuting flows.

Each distinct function's geographical distribution reveals a core-periphery structure with central business districts, surrounding residential zones, and secondary commercial centers. This pattern aligns with established urban planning theory [1, 2]. The identification of these distinct functional zones is a robust finding; as shown in Supplement Fig S8, smaller cities like Fukuoka, Sapporo, and Sendai exhibit similar, albeit more isolated, core-periphery structures, highlighting universal patterns of urban density and connectivity across different metropolitan scales.

3. Robustness of Commuting Time Metrics (Eq. 5): While the authors use the minimum commuting time among observed trips, I suggest conducting a robustness check using alternative measures (e.g., average, median commuting times) to verify that the choice of metric does not unduly affect the model's performance. Additionally, it would be valuable to discuss why commuting times can vary for the same OD pair, especially since the data indicate actual variability in these times.

Response:

We thank the reviewer for this insightful suggestion to test the robustness of our commuting time metric and to elaborate on the sources of its variability. This prompted us to conduct an important validation that has ultimately strengthened our manuscript.

Robustness Check

As suggested by the reviewer, we conducted a robustness check by recalibrating our model with the mean and median commuting times. The results, shown in Fig. 2, confirm that the model's structure is robust, as it produces reasonable predictions with all three metrics.

This outcome is consistent with the statistical properties of the commuting data. The mean is highly sensitive to outliers—unusually long trips caused by traffic or detours—which explains its lower accuracy. Mathematically, given that the commuting times follow an exponential distribution (as shown in Supplement S3), the probability mass is heavily concentrated on shorter trips. Both the median and minimum are robust measures that capture this central tendency effectively. The minimum time, in particular, likely represents the ideal, uninfluenced travel cost between two points, providing the most stable and accurate deterrence factor for the model.

This content as been appended in Supplement Section 8, also in the manuscript, we added: line 313: Robustness tests for this assumption, where minimum values are replaced with mean and median values, are presented in the Supplement.

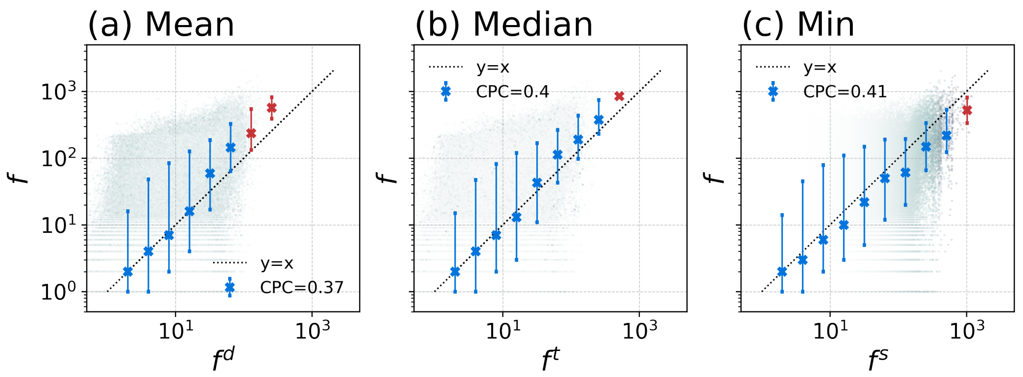

Figure 2: Robustness test results comparing model performance using minimum, mean, and median commuting times

Sources of Commuting Time Variability

We agree with the reviewer that a discussion on why commuting times vary for the same OD pair is valuable. This variability is a key feature of real-world mobility and stems from several factors, which we now discuss in the revised manuscript line 297: However, commuting time is dependent on the behavior of individual commuters, meaning that each OD pair can contain multiple commuting times. This real-world variability stems from several factors:

- Transportation Mode: For a single OD pair, individuals may use various modes with different time profiles, such as private cars, motorcycles, bicycles, or different public transit options (e.g., local vs. express trains).
- Route and Service Choice: Even within a single mode, choices matter. Drivers may choose between highways and local roads, while transit users may select different lines or transfer points.
- Real-time Conditions: Road traffic congestion is a major source of variability for drivers. Similarly, public transit is subject to delays, crowding, and service schedules that vary by time of day.
- Individual Behavior: Raw trajectory data can capture non-commuting detours, such as stopping for errands, which increase total travel time.

Despite this variability, we assume that the fundamental deterrence for an OD pair is best represented by the shortest possible travel time...

4. Statistical Validity of F-Test Application (Eq. 10): The authors use an F-test to assess the significance of introducing segmentation in the gravity model. However, Eq. (10) assumes that parameters are estimated via OLS under normally distributed errors, whereas the manuscript indicates that parameters are estimated using Bayesian optimization (BO). Could the authors clarify the validity of applying the F-test in this context? Additional explanation in Appendix A5.2 is warranted.

Response:

We thank the reviewer for this critical question regarding methodological consistency. We acknowledge the apparent discrepancy and have revised the text to clarify the distinct roles of the F-test and Bayesian Optimization in our study.

The F-test was not used to fit or evaluate the final SSUG model. Rather, it was employed as a preliminary, exploratory analysis on a simplified log-linear version of the gravity model (Eq. 7). The

purpose of this test was solely to statistically validate our core hypothesis: that there is a significant structural break in the data, justifying the segmentation of population scales.

Once the necessity of segmentation was statistically established, we then employed Bayesian Optimization to calibrate the full, non-linear SSUG model (Eq. 4). BO is far better suited for this complex, eight-parameter optimization problem for which simple linear regression is inadequate.

We have amended Manuscript Sections 2.3 and Appendix A.5.2 to make this two-stage approach explicit and to clarify that the F-test serves as a justification for the model structure, not as the final parameter estimation method.

In Manuscript Section 2.3, line 351: ...Specifically, we employed F-tests to statistically validate our core hypothesis that structural breaks exist at the thresholds h_c and w_c. The F-test determines whether the relationship between population variables (h_i or w_j) and commuting flows (f_{ij}) differs significantly for values below versus above each threshold. A significant F-statistic indicates that the data should indeed be treated as two distinct regimes, thereby justifying our segmented modeling approach based on population scale thresholds.

It is important to note that these threshold validation tests serve solely to confirm the existence of structural breaks in the data—the parameters derived from these F-tests were not incorporated into the final SSUG model fitting or evaluation procedures described below.

We add Appendic A.5.3 after A.5.2 for this justification:

A.5.3

To rigorously test this hypothesis, we first conducted a preliminary statistical analysis to determine whether dividing the dataset into two populations based on numerically defined population scale thresholds results in significantly different gravity model parameters.

- **Baseline specification.** We first estimate the conventional log-linear gravity model,

$$\log F_{ij} = \alpha + \beta_1 \log H_i + \beta_2 \log W_j + \gamma \log t_{ij} + \varepsilon_{ij},$$

  by ordinary least-squares (OLS).

- **F-test on population splits.** Because the model is linear in logs, OLS residuals allow a classical F-test (ANOVA) to check whether the coefficient vector differs across population-density groups.

- **Scanning all candidate thresholds.** We sweep every integer value of the residential and workplace population cut-offs (h, w) within the observed range (see Fig. 1). For each split we (i) re-estimate the log-linear model in the two sub-samples and (ii) compute the F-statistic against the pooled model. The assumptions of the test—normality and homoscedasticity of residuals hold throug centain ranges.

- **Rationale for Bayesian optimisation (BO).** Once the F-test confirms that *some* statistically significant segmentation exists, we embed the cut-offs as continuous parameters (h_c, w_c) in the nonlinear SSUG formulation and let Bayesian optimisation jointly estimate (h_c, w_c) together with the eight segment-specific coefficients, yielding fully data-driven thresholds.

5. CPC Evaluation Metric: The manuscript currently uses the CPC index to assess model fit. However, since the SSUG model is calibrated via Bayesian optimization (BO), it may be simpler and more consistent to use the value of the BO objective function directly as the evaluation metric, rather than introducing an additional index. This approach would ensure coherence between the evaluation measure and the model calibration process.

Response:

We thank the reviewer for this thoughtful question regarding the consistency between our optimization function and evaluation metric. We agree this is an important point and have revised the manuscript to provide greater clarity and transparency.

We wish to clarify the distinct roles of the objective function and the CPC index in our study:

- **Loss Function for Optimization:** The objective function defined in Manuscript Eq. 6 was selected specifically as a loss function to guide the Bayesian Optimization process. Its logarithmic form is mathematically well-suited for handling flow data that spans several orders of magnitude, ensuring a stable and efficient search for the optimal model parameters. However, the final numerical value of this function is not directly interpretable in a physical sense.
- **Evaluation Metric for Communication:** The CPC index was chosen as our primary evaluation metric for its clear interpretability and its established use in the human mobility literature. As a normalized index ranging from 0 to 1, the CPC provides an intuitive measure of how well our model's predictions overlap with empirical data, which is crucial for communicating our findings and for benchmarking our model against previous and future research.

Because the three models (the SSUG model, Time-based Gravity model, Distance-based Gravity model) use different optimization methods, a direct comparison of these raw objective function values can be misleading. We believe the CPC index is a relatively correct and necessary tool for a fair, standardized comparison of the models' predictive performance.

We agree with the reviewer that providing the value of the objective function adds transparency. Therefore, in the spirit of this suggestion, we have now added the final optimized value of the objective function to our main results in Supplement Table 6 as below.

13 Objective Function Values

The final values of the objective function (defined in the manuscript's Eq. (6)) for each of our tested models.

Model	Objective Function Value
SSUG Model	-0.1290
Time-based Gravity Model	-7.81×10^{-12}
Distance-based Gravity Model	-1.23×10^{-14}

Table 1

As shown in Table 1, since we optimized the model by minimizing the objective function, the lowest (most negative) value of -0.1290 corresponds to the best performance. Therefore, according to the defined optimization goal, the SSUG model achieved the best result, followed by the Time-based Gravity model, with the Distance-based Gravity model performing least effectively.

References

[1] Xu Y, Olmos LE, Mateo D, Hernando A, Yang X, González MC. Urban dynamics through the lens of human mobility. Nature Computational Science. 2023;3:611–620. doi:10.1038/s43588-023-00484-5.

[2] Fanelli F, Melo HPM, Bruno M, Loreto V. Revealing the core-periphery structure of cities; 2024. Available from: https://arxiv.org/abs/2410.21133.
