# Supplementary material for: Enhancing the gravity model for commuters: Time-and-spatial-structure-based improvements in Japan’s metropolitan areas
Source: PLoS One. 2025 Aug 12;20(8):e0329603. doi: 10.1371/journal.pone.0329603 (PMC12342275; doi:10.1371/journal.pone.0329603)
Supplement: Attachment — Submitted filename: report.pdf [file pone.0329603.s006.pdf]

Referee Report on "Enhancing the Gravity Model for Commuters: Time-and-Spatial-Structure-Based Improvements in Japan's Metropolitan Areas" by Yixuan Y Zheng, Yohei Shida, Hideki Takayasu, and Misako Takayasu. (PONE-D-24-49532R2)

I appreciate the authors' efforts to address the concerns raised in the first round of review. The revisions provided generally respond appropriately to the previous comments. I find that all responses except for the one regarding Comment 1 are satisfactory.

Regarding Comment 1 (Constraint 1):

The authors assert that when $h_i = 1$ and $w_j = 1$, it must follow that $f_{ij} = 1$. While this assumption is internally consistent under a one-to-one assignment of commuters, it becomes problematic when there are multiple zones with one resident or one worker.

To illustrate, consider the following scenario:

- $h_i = 1$ (zone i has one resident)
- $w_k = 1$, $w_j = 1$ (zones k and j each have one job)
- $f_{ik} = 1$ and $f_{ij} = 1$, as implied by Constraint 1

In this case, the total outflow from zone i would be 2, which violates the condition $h_i = 1$. Therefore, Constraint 1 cannot simultaneously hold for both (i, j) and (i, k). More broadly, if $h_i = 1$ and $w_j = 1$, then it must also be the case that $w_k \neq 1$ for all $k \neq j$, and $h_k \neq 1$ for all $k \neq i$. This indicates that the data vectors h and w must each contain *at most one entry equal to 1* for Constraint 1 to remain consistent.

I encourage the authors to explicitly clarify this exclusivity assumption in the manuscript. Without it, the use of Constraint 1 as a basis for normalization could conflict with flow conservation and undermine the model's internal consistency.
