# Supplementary material for: Enhancing the gravity model for commuters: Time-and-spatial-structure-based improvements in Japan’s metropolitan areas
Source: PLoS One. 2025 Aug 12;20(8):e0329603. doi: 10.1371/journal.pone.0329603 (PMC12342275; doi:10.1371/journal.pone.0329603)
Supplement: Attachment — Submitted filename: Response_to_Reviewers_auresp_3.pdf [file pone.0329603.s007.pdf]

Response to Editor and Reviewers

Dear Editor and Reviewers,

We are grateful for the opportunity to submit a second revision of our manuscript and sincerely appreciate the constructive feedback provided during the review process. The additional comments have been invaluable in helping us strengthen the methodological rigor and clarity of our work.

To the Editor: We extend our sincere appreciation for your expert management of the review process and for facilitating the constructive dialogue between authors and reviewers. We are grateful for your patience and professional oversight during the revision process.

To Reviewer 4: We thank you for your diligent and detailed second review. We agree that your remaining comment regarding the logical consistency of Constraint 1 is a critical point. In response, we have focused this revision on addressing this issue directly. As detailed below, we have clarified the data preprocessing rule that ensures the validity of this constraint, thereby resolving the potential inconsistency you identified.

To Reviewer 3: We extend our heartfelt thanks for your positive assessment and recommendation for publication. Your supportive evaluation of our manuscript is greatly appreciated.

We believe these final revisions substantially enhance the manuscript's methodological rigor and address the remaining concerns raised during the review process. The specific changes and detailed explanations are outlined in the comprehensive response that follows.

Response to Reviewer 4

Regarding Comment 1 (Constraint 1):

The authors assert that when $h_i = 1$ and $w_j = 1$, it must follow that $f_{ij} = 1$. While this assumption is internally consistent under a one-to-one assignment of commuters, it becomes problematic when there are multiple zones with one resident or one worker.

To illustrate, consider the following scenario:

- $h_i = 1$ (zone i has one resident)
- $w_k = 1$, $w_j = 1$ (zones k and j each have one job)
- $f_{ik} = 1$ and $f_{ij} = 1$, as implied by Constraint 1

In this case, the total outflow from zone i would be 2, which violates the condition $h_i = 1$. Therefore, Constraint 1 cannot simultaneously hold for both (i, j) and (i, k). More broadly, if $h_i = 1$ and $w_j = 1$, then it must also be the case that $w_k \neq 1$ for all $k \neq j$, and $h_k \neq 1$ for all $k \neq i$. This indicates that the data vectors h and w must each contain *at most one entry equal to 1* for Constraint 1 to remain consistent.

I encourage the authors to explicitly clarify this exclusivity assumption in the manuscript. Without it, the use of Constraint 1 as a basis for normalization could conflict with flow conservation and undermine the model's internal consistency.

Response:

We sincerely thank the reviewer for this insightful comment. The reviewer is entirely correct to point out that Constraint 1, as stated, could appear inconsistent if the input data were not handled carefully. The hypothetical scenario presented—where a single resident from zone i ($h_i = 1$) is simultaneously assigned to two different single-job zones ($w_j = 1$ and $w_k = 1$)—would indeed violate the flow conservation principle ($\sum_j f_{ij} = h_i$).

We are grateful for this opportunity to clarify that this exact issue is prevented by a crucial data preprocessing step mentioned in our original manuscript. As the reviewer astutely noted, our assumption about how to handle commuters with multiple workplaces is strongly correlated with the validity of Constraint 1. Our response was implicit in our data preparation, and we agree that it must be made explicit.

First, as we stated in the original manuscript in Section 2.1.1 Residential and workplace identification (line 189):

For users who might have more than one workplace or whose workplace is artificially divided by grid boundaries (approximately 3%), we used the first workplace visited after leaving home to capture accurate commuting distance and time (Section 2.1.2).

This 'first workplace' rule is the key. It ensures that any single commuter contributes to the job count (w) of only one zone. Therefore, the paradoxical situation the reviewer describes is methodologically prevented from occurring in our model's input data. A single resident in zone i cannot be the source for a count of $w_j = 1$ and $w_k = 1$.

We have expanded upon our initial statement in Section 2.1.1. In line 189, we have added the following clarification to emphasize the implication of our assumption:

This assumption is significant for our model as it ensures that each commuter is uniquely assigned to one home and one workplace. This one-to-one mapping at the individual level is fundamental for the internal consistency of the gravity model-based approach, preventing a single commuter from being counted as an outflow to multiple destinations (Section 2.2).

Furthermore, we have clarified the explanation of Constraint 1 to incorporate this logic directly. Following the illustration of Constraint 1, in line 255, we have revised it as follows:

Constraint 1 states that when an origin i has one commuter ($h_i = 1$) and a destination j also has one job ($w_j = 1$), the observed flow between them must be one ($f_{ij} = 1$). The consistency of this constraint is guaranteed by our preprocessing rule for handling multiple workplaces, as detailed in Section 2.1.1. For instance, in the scenario where a single resident from zone i might travel to two potential single-job zones, j and k, our methodology resolves the ambiguity as follows:

- A single resident is located in zone i ($h_i = 1$).
- This resident first travels to a workplace in zone j and later to a workplace in zone k.
- Per our rule, only the first workplace is considered. The input data for the model is therefore processed as $w_j = 1$ and the contribution to w_k from this resident is 0.
- Consequently, the model correctly enforces $f_{ij} = 1$ and $f_{ik} = 0$, preserving the flow constraint that the total outflow from zone i is 1.

This ensures our model remains internally consistent and that Constraint 1 provides a valid baseline for normalization. It also provides a consistent baseline for the constant $K(K_1)$ across all four model types, ensuring continuity and model interpretability as shown below.

Together, these constraints transform our initial formulations into more complex expressions with consistent behavior at boundary conditions (for clarification, please see Supplement Section 11).

We believe these revisions directly address the reviewer's valid concern, clarifying that our model's consistency is maintained not by placing restrictive conditions on the data (e.g., that vectors h and w can only have one entry equal to 1), but by a clear and explicit rule applied during data preparation. We thank the reviewer again for prompting this important clarification.
